# A genome-wide association study using international breeding-evaluation data identifies major loci affecting production traits and stature in the Brown Swiss cattle breed

**DOI:** 10.1186/1471-2156-13-82

**Published:** 2012-10-02

**Authors:** Jiazhong Guo, Hossein Jorjani, Örjan Carlborg

**Affiliations:** 1 College of Animal Science and Technology, Northwest A&F University, Yangling, Shaanxi, 712100, People’s Republic of China; 2 Department of Clinical Sciences, Division of Computational Genetics, Swedish University of Agricultural Sciences, Uppsala, Sweden; 3 Department of Animal Breeding and Genetics, Swedish University of Agricultural Sciences, Uppsala, Sweden

## Abstract

**Background:**

The genome-wide association study (GWAS) is a useful approach to identify genes affecting economically important traits in dairy cattle. Here, we report the results from a GWAS based on high-density SNP genotype data and estimated breeding values for nine production, fertility, body conformation, udder health and workability traits in the Brown Swiss cattle population that is part of the international genomic evaluation program.

**Result:**

GWASs were performed using 50 k SNP chip data and deregressed estimated breeding values (DEBVs) for nine traits from between 2061 and 5043 bulls that were part of the international genomic evaluation program coordinated by Interbull Center. The nine traits were milk yield (MY), fat yield (FY), protein yield (PY), lactating cow’s ability to recycle after calving (CRC), angularity (ANG), body depth (BDE), stature (STA), milk somatic cell score (SCS) and milk speed (MSP). Analyses were performed using a linear mixed model correcting for population confounding. A total of 74 SNPs were detected to be genome-wide significantly associated with one or several of the nine analyzed traits. The strongest signal was identified on chromosome 25 for milk production traits, stature and body depth. Other signals were on chromosome 11 for angularity, chromosome 24 for somatic cell score, and chromosome 6 for milking speed. Some signals overlapped with earlier reported QTL for similar traits in other cattle populations and were located close to interesting candidate genes worthy of further investigations.

**Conclusions:**

Our study shows that international genetic evaluation data is a useful resource for identifying genetic factors influencing complex traits in livestock. Several genome wide significant association signals could be identified in the Brown Swiss population, including a major signal on BTA25. Our findings report several associations and plausible candidate genes that deserve further exploration in other populations and molecular dissection to explore the potential economic impact and the genetic mechanisms underlying these production traits in cattle.

## Background

Genome wide association studies (GWAS) have for a number of years been a useful tool for detecting genetic variants associated with complex traits in human genetics [1,2]. With the advancement of genotyping and sequencing technology, platforms for high-density genotyping have been developed for other species as well – a development that has facilitated GWAS also in e.g. livestock [3-7], domesticated plants [8] and model organisms [9].

Since the seminal QTL mapping work in cattle by Georges et al. [10], a large number of studies have reported QTL for many different traits in various breeds. The limitation of QTL mapping in identifying the causal variants underlying the studied traits using the low to moderate dense marker maps have been discussed in depth elsewhere [11,12]. In contrast to traditional QTL mapping strategies, GWAS opens new opportunities to make efficient use of outbred cattle populations for high-resolution mapping of loci of even modest effects underlying important production traits [13]. A main motivation for performing GWASs in domesticated animals, like dairy cattle, is thus to discover genes, or potentially even causal mutations, contributing to the phenotype of economically important traits. Such findings could be important for improving the accuracy of breeding value estimation and also to increase our understanding of the mechanisms underlying long-term selection response in artificial breeding program.

A distinct feature of dairy cattle populations is the recent small effective population size that results from a widespread use of artificial insemination. Furthermore, dairy cattle have been subjected to long-term, intensive directional artificial selection and assortative mating scheme for milk production traits in general, and milk yield in particular. This population history has largely influenced the pattern of linkage disequilibrium (LD) in the dairy cattle breeds. For instance, the Bovine Hapmap Consortium [14] reported low, but nonzero, levels of LD at distances of up to 1 Mb in several dairy cattle breeds. As GWAS analyses exploit LD, it is therefore possible to perform powerful genome-wide analyses in dairy cattle with a marker density much lower than that in humans, i.e. using markers positioned every 100 kb or so [15]. Recent GWAS studies in dairy cattle have primarily focused on the Holstein breed [3-5,16-18], due to its widespread use across the world. Some studies have also been published in other breeds, including one of direct gestation length in Brown Swiss cattle [19].

A major challenge in the statistical analysis of GWAS data is the sensitivity to systematic confounding factors that might lead to false positive associations, and the primary causes of such confounding result from either population stratification or family structure [20]. So far, several different methods to correct for systematic confounding have been proposed [21-23]. However, due to the multiple families and multiple generations in livestock GWAS datasets, the systematic confounding is rather complex. Linear mixed models have been suggested as a way to effectively correct for subpopulation and/or family structure in order to reduce the rate of false positives without too much loss in power [24-27], which makes this method the approach of choice in such situations.

The Brown Swiss cattle is best known for their high milk yield and ability to produce well under challenging conditions in terms of temperature and feed. It is important in dairy production in Western Europe and North America. Since 1996, the Interbull Centre has routinely received data from Brown Swiss bulls from many countries and carried out an international breeding evaluation resulting in comparable estimated breeding values (EBVs) for these bulls across countries. In this study, we make use of the international EBVs of Brown Swiss bulls from several countries and genotypes from the Illumina Bovine SNP50 Beadchip (Illumina Inc., San Diego, USA) that were collected for use in the national and international genomic prediction effort, to perform a GWAS to identify loci contributing to several for economically important traits in Brown Swiss cattle.

## Results

### Description of evaluated phenotypes

The nine traits selected for this GWAS analysis were milk yield (MY), fat yield (FY), protein yield (PY), lactating cow’s ability to recycle after calving (CRC), angularity (ANG), body depth (BDE), stature (STA), milk somatic cell (SCS) and milking speed (MSP). The descriptive statistics of these traits are provided in Table 1. According to the convention for classification of traits at the Interbull Centre, the investigated traits can be classified into five classes: production-, fertility-, conformation-, udder health- and workability traits. In total, genotypes were available for 7038, but here we only included the progeny proven bulls in the GWAS analysis. As the start of the national genetic evaluations, and the measurement time for the traits of daughters differed, the final sample sizes in the GWAS analysis varied between 2061 and 5043 for the traits (Table 1). As the bull’s international breeding values were based on both pedigree information and daughter phenotypes, we used a conversion equation to transform EBVs to deregressed-EBVs (DEBVs) to remove the contributions of information from relatives. The DEBVs were then used as the phenotypes in the analysis. The milk production traits had high heritability and the EBVs for these traits were very accurate. Both the reliability and heritability were lowest for the fertility trait.

**Table 1:** Descriptive statistics of the phenotypes (deregressed EBVs) used in the study

**Trait**	**Trait type**	**Mean**	**SD**	**n**	**Mean reliability**^**a**^	**h**^**2**^
MY	Production	−0.79	1.88	5043	0.809	0.360
FY	Production	−0.82	1.75	5043	0.802	0.340
PY	Production	−0.95	1.70	5043	0.790	0.350
CRC	Fertility	0.11	1.36	3807	0.643	0.039
ANG	Conformation	0.35	4.19	2061	0.731	0.300
BDE	Conformation	−0.14	1.38	4402	0.741	0.372
STA	Conformation	−0.45	1.33	4610	0.824	**0.428**
SCS	Udder health	−0.27	1.24	4803	0.797	0.270
MSP	Workability	−0.16	1.19	4411	0.758	0.144

a: reliability of EBV values.

The pairwise Pearson correlation coefficients for all pairs of traits are given in Table 2. The milk production traits are strongly and positively correlated, but also traits within and between the other categories often had intermediate correlation. The only trait that is only mildly correlated to other traits is milking speed.

**Table 2:** Pairwise Pearson phenotypic correlations

**Trait**	**MY**	**FY**	**PY**	**CRC**	**ANG**	**BDE**	**STA**	**SCS**
**FY**	0.891							
**PY**	0.893	0.921						
**CRC**	−0.315	−0.295	−0.269					
**ANG**	−0.332	−0.332	−0.325	0.109				
**BDE**	0.415	0.391	0.369	−0.115	−0.336			
**STA**	0.269	0.281	0.360	−0.091	−0.450	0.551		
**SCS**	0.152	0.193	0.174	0.101	0.007	−0.101	0.009	
**MSP**	−0.009	0.049	0.095	−0.101	−0.187	−0.128	0.033	−0.109

### Estimation of population stratification using principle component analysis

To examine the possible population structure in the analyzed population, principal component analysis (PCA) was performed for all 7038 individuals based on the kinship matrix estimated from SNPs. Two sub-groups were identified of 6784 and 254 bulls, respectively (see Additional file 1: Figure S1). The total amount of genetic variance explained by the first two principal components was, however, low (12.01% and 1.68%), indicating that this stratification is unlikely to have any strong influence on the outcome of the GWAS analysis. This was also confirmed by further exploratory analyses showing that analyzing the two sub-classes separately produced similar results to those when the whole population was analyzed jointly (results not shown).

### Genome-wide association analysis accounting for confounding

**Figure 1:**
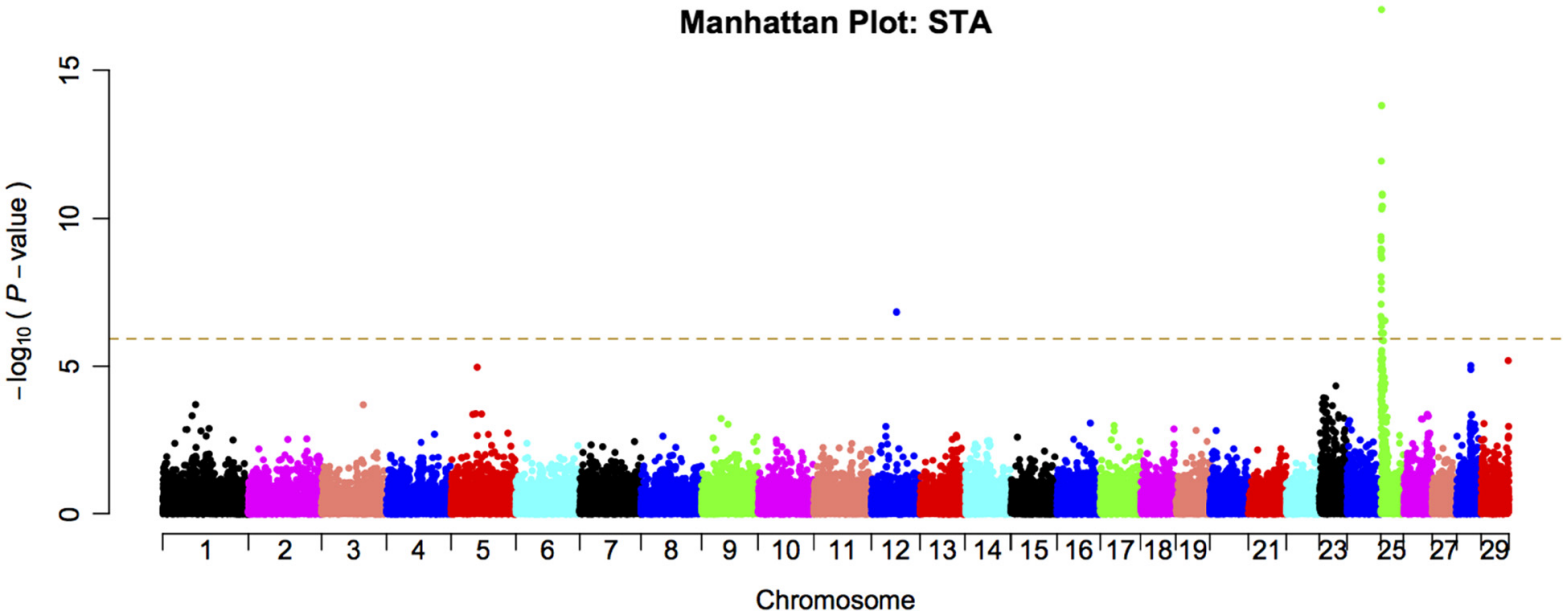
**Manhattan plots for stature based on a linear mixed model and genomic control.** Manhattan plot showing the association of stature to BTA1-29 with the chromosomes plotted separately by color. The *p*-values of the association test were after genomic control transformed to –log10 (*p*-values). The horizontal dashed line indicates the genome-wide Bonferroni-corrected significance level.

The data was analyzed using a linear mixed model, as implemented in EMMAX [26], and the results corrected using genomic control [21]. The manhattan plots of the obtained *P*- values are given in Figure 1 (stature) and Additional file 2: Figure S2 (the other 8 traits) (see Additional file 2: Figure S2). A total of 74 genome-wide significant SNPs were identified for these nine traits (Table 3). The association signals were distributed across 12 chromosomes, with the strongest signals on BTA25. Some SNPs were significant for multiple traits and the number of uniquely significant SNPs was 50. As shown in the quantile-quantile (Q-Q) plots for the GWAS analyses (see Additional file 3: Figure S3), the *P*-value inflation was low, but with λ –values still significantly larger than 1. However, the λ_1000_ values were close to 1.05, which is generally considered benign [20]. The estimated effect and *P*-values of all the significant SNPs for the analyzed traits are listed in Table S1 (Additional file 4: Table S1).

**Table 3:** Numbers and distribution of genome-wide significant SNPs detected by EMMAX with genomic control

**Trait**^**a**^									
**BTA**	MY	FY	PY	CRC	ANG	BDE	STA	SCS	MSP
1					1				
5	1	1							
6									2
8					1				
11					5				
12					1		1		
17					1				
20	1	1				1			
24	1							1	
25	2	5	3	4		12	26		
28	1								
29					2				
Total	6	7	3	4	11	13	27	1	2

^*a*^*MY*: milk yield, *FY*: fat yield, *PY*: protein yield, *CRC*: lactating cow’s ability to recycle after calving, *ANG*: angularity, *BDE*: body depth, *STA*: stature, *SCS*: milk somatic cell count, *MSP*: milking speed.

**Figure 2:**
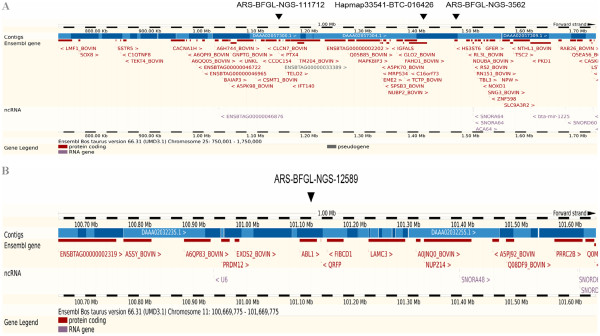
**1 Mb gene clusters surrounding the association signals for stature and production traits on BTA25 and angularity on BTA11.**Gene clusters in 1 Mb regions surrounding the main association signals for **A**) stature and production traits on BTA25 and **B**) angularity on BTA11.

The peaks for stature, milk yield, fat yield, protein yield, lactating cow’s ability to recycle after calving and body depth covered almost the same region on BTA25. The most significant SNP and the number of significant SNPs did, however, vary. Based on the results from the linear mixed model analysis with subsequent genomic control, the number of genome-wide significant SNPs at the peak on BTA25 for stature, milk yield, fat yield, protein yield, lactating cow’s ability to recycle after calving and body depth was 26, 2, 5, 3, 4 and 11, respectively. The 26 associated SNPs with stature covered a 7.65 Mb interval from 32 kb to 7.95 Mb. For body depth, the 11 genome-wide significant SNPs covered 1.7 Mb from 1.1 to 2.9 Mb. A number of genes mapped to the short region closely surrounding the most significant SNPs, including functional candidate genes including *insulin-like growth factor binding protein acid labile subunit* (*IGFAL), hydroxyacylglutathione hydrolase* (*HAGH*)*,* and *heparan sulfate (glucosamine) 3-O-sulfotransferase 6* (*HS3ST6*) (Figure 2A). A second less significant peak with only one significant SNP was also observed for body depth at 33 Mb on BTA25. In addition, milk yield and fat yield shared one associated SNP at 33.17 Mb on BTA5 and another one at 63.12 Mb on BTA20 (see Additional file 4: Table S1.1, Table S1.2).

A peak consisting of five SNPs with genome-wide significant effects on angularity was detected at the distal region of BTA11. Four of these five SNPs were located between 87.3 Mb and 88.0 Mb. The closest gene mapping to this peak is the *c-abl oncogene 1* (*ABL1*) (Figure 2B), but other genes including *pyroglutamylated RFamide peptide* (*QRFP*), *fibrinogen C domain containing 1* (*FIBCD1*), *laminin gamma 3* (*LAMC3*) were close to the signal. Other SNPs associated with angularity were also detected on BTA1, 8, 12 and 29.

Several SNPs affecting somatic cell score were located in a peak on BTA24, although only one of these (located at 31.1 Mb) reached genome wide significance. An interesting candidate microRNA (*bta-mir-2380*) was located close to the peak at 31.1 Mb (see Additional file 5: Figure S4A).

A signal associated with milking speed was identified on BTA6 and two SNPs, at 90.3 and 90.5 Mb, reached the genome-wide significance level. Here, the *afamin* (*AFM* or *ALB2*), *alpha-fetoprotein* (*AFP or FETA_Bovine*), *albumin* (*ALB*) as well as the Interleukin-8 (IL8) genes were located in the close vicinity of the peak (see Additional file 5: Figure S4B).

### Genome-wide association analysis for production traits conditional on stature

**Figure 3:**
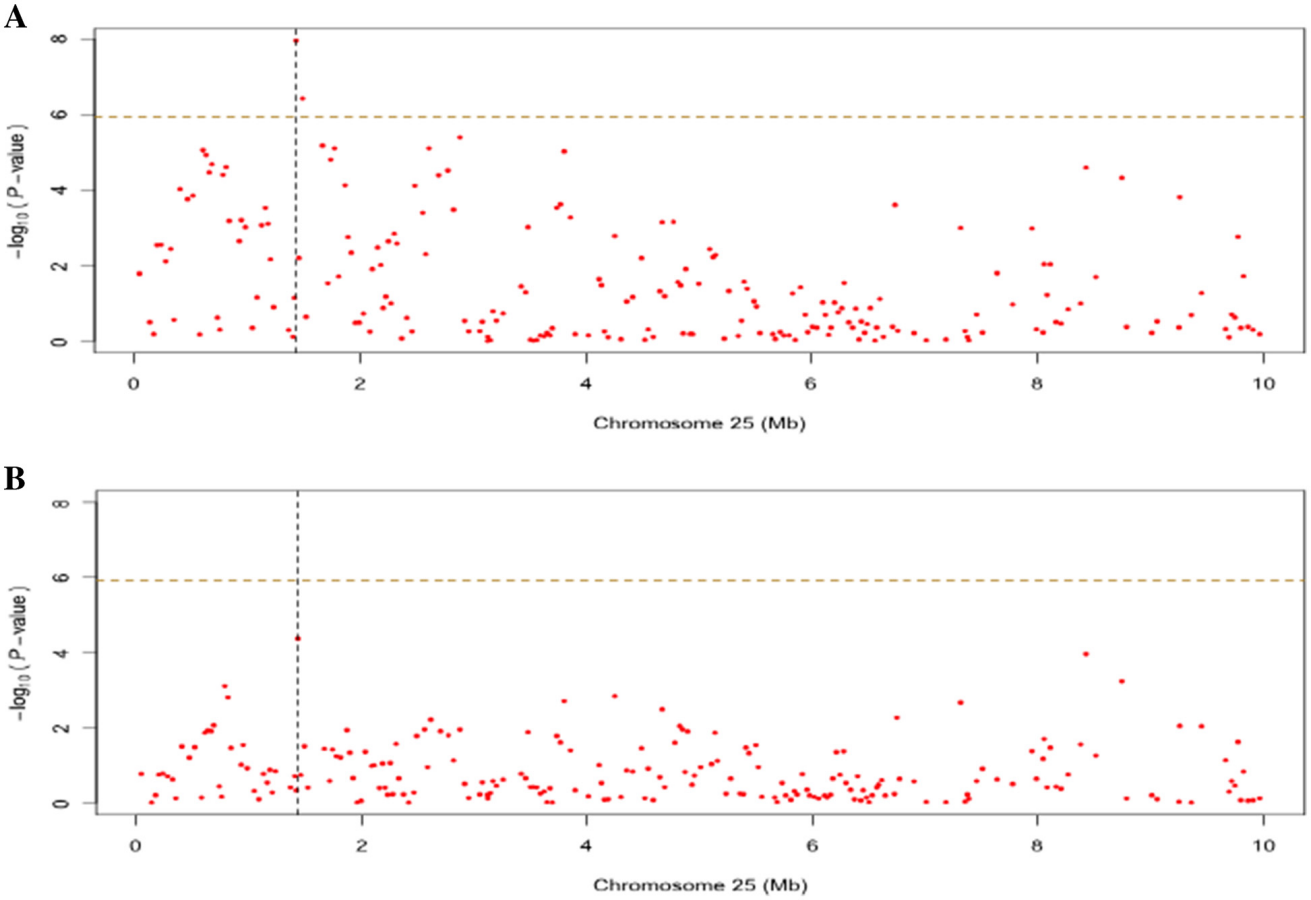
**The association of protein yield to BTA25 with and without stature as a covariate.** The *p*-values for the association of protein yield to BTA25 was obtained using a linear mixed model and genomic control and then transformed to –log10 (*P*-values). The horizontal dashed line indicates the genome-wide significance level (i.e. corrected for testing all markers on BTA1-29). Figure 3A shows the result of the analysis without and 3B the analysis with the stature phenotype as a covariate in the linear model.

In order to evaluate the potential pleiotropy of the association peak on BTA25 that affected multiple traits, additional GWAS analyses were performed for the production traits using a linear mixed model, where the stature phenotype was included as a covariate. The results from these analyses show a decrease, although not a complete removal, of the peak surrounding the most significant SNP on BTA25. One SNP for fat yield and protein yield still, however, reached the genome-wide significance indicating that the effect on the production traits is not necessarily a secondary effect of the size of the animal, and a pleiotropic locus with effects on both stature and production might be present (Figure 3; see Additional file 6: Figure S5; see Additional file 7: Figure S6).

## Discussion

### Population structure and GWAS

Here, we made use of the data available from the international genetic evaluation of dairy cattle at Interbull Centre. In this way, we could include most of the Brown Swiss progeny proven bulls from seven countries in a GWAS to obtain a large, powerful sample for detecting genetic variants associated with economically important dairy cattle traits. From a population genetics point of view, this population represents a major part of the paternal genetic material in the contemporary global Brown Swiss population. Given the large population size, this study is expected to provide useful insights to important loci under selection for the main production traits within the Brown Swiss cattle population. To date, no GWAS study on milk production traits and stature in Brown Swiss population has been published, making these results of greater interest as they will allow comparisons with results on studies of other dairy breeds.

Our GWAS study is a population-based study and is then potentially sensitive to population admixture and familial relatedness caused by recent selection and/or non-random mating. It is well known that these systematic factors could lead to spurious association results, and different approaches correcting for these have been proposed and discussed [21-23]. In this study, we first used PCA analysis to examine whether population structure could be of major concern. The results indicate that the sample of Brown Swiss bulls could be divided into two groups – one large and one small. Compared to other PCA analysis results in structured populations, however, the first and second principal components here explained a much smaller proportion of the total genetic variation. Even though the genomic kinship indicated that the population could be divided into two groups, the overlap with the pedigree structure was small. Most members of the smaller group of bulls originated from Switzerland. We hypothesized that these might be from the original Brown Swiss cattle. However, a PCA analysis of all Brown Swiss cattle from Switzerland (Bapst, personal communication) showed that there is no overlap between the old Brown Swiss cattle and the smaller group in our study. Our interpretation is that the apparent confounding is a false positive result that likely arose in the PCA analysis [28]. As these results suggested that the population structure was not of main concern, we did not exclude the individuals from the smaller group in the final GWAS analysis.

Unlike the normal pattern of confounding factors in human GWAS studies, familial relationship under intensively directional artificial selection is the chief confounding factor in domesticated animal GWAS studies. This can potentially result in genome-wide inflation of the *P*-values in the GWAS and result in false positives in the same way as population stratification. When high familial relatedness exists, but only slight population stratification, linear mixed models are useful for performing powerful association analyses, while at the same time reducing the false positive rate [24-27]. In these analyses, a polygenic random effect term is included to represent the family structure. In our study, the analyses were performed using EMMAX, and the first set of results indicated that an intermediate or small inflation still occurred. There are several potential explanations for this. First, it might be caused by the impact of strong artificial selection influencing a smaller subset of loci, which then will be different from the general impact of subpopulation divergence on the whole genome. Such inflation will differ between different types of traits. Our results from a simple regression analysis of the data are in line with this explanation, as the inflation varied greatly between different types of traits in these analyses (data not shown). Secondly, when a number of loci have strong effects on the traits, the overall inflation of the *P*-values might be affected by some strong signals in the upper tails of the *P* values. This is something observed here, where for most traits were the strongest deviations from the expected *P*-values under the null-hypothesis is observed in the upper tail of the distribution. Also, the inflation was larger for production traits than for the other traits. As Brown Swiss cattle, in the same way as other dairy cattle, have been most strongly selected for milk production traits, the difference in inflation between the traits could reflect the genomic influence of selection on a limited number of major loci.

### Comparison with reported results and pleiotropy for production and body size traits

As the number of GWAS studies in cattle is still limited, we have made an attempt to overlap our association signals with those of previously reported QTL. Although the complete and exact translation of bovine genetic distances into physical distances is not available, we used the information of the physical map location provided in the cattle QTLdb in animal genome database [29] to do these comparisons.

In our study, the main association was found on BTA25. Most of the significant SNPs were located around 1.1-1.4 Mb and affected stature, milk yield, fat yield, protein yield, lactating cow’s ability to recycle after calving and body depth. A second peak was mapped for body depth at around 33 Mb. Several earlier studies have reported overlapping QTL for milk- and protein yield on BTA25 at around 32.9- 44.0 Mb (45–69 cM) in Holstein and Finnish Ayrshire [3,29-32]. This peak thus overlapped with our association signal for body depth. No earlier milk production QTL has been reported for our major region. Given the major effect of our locus on stature it is interesting, however, to note that recently reported QTL influencing body weight in an Angus population [33] was located in this region (79 kb-13.3 Mb or 0.6-14 cM). Given that stature, body depth and body weight are all measures of body size, it is possible for them to be sharing underlying pathways and causal variants. Indeed, Cole et al. [18] report that some associated SNPs were shared by the two traits in US Holstein population. The most interesting candidate gene in this region in the NCBI and ENSEMBL databases [34,35] is *IGFAL*. This gene is a serum protein that binds IGFs that regulate growth, development and other physiological process. It interacts with the growth hormones and increases their half-life and their vascular localization [36]. Courtland et al. [37] have earlier reported that sex-specific effects of body- and bone size depended on *IGFAL.* Furthermore, *IGFAL* is also known to be associated with growth deficiencies in human [38], which further suggests *IGFAL* as a major candidate gene for these growth related traits [39]. Based on the GWAS analysis for production traits conditional on the phenotypes of stature, it is reasonable that the alleles at this locus have a pleiotropic effect on the growth and milk production traits. Although part of the difference in production could be explained by the basic biological logic that bigger body size leads to higher milk yield, our results indicate that this locus also has direct effects on milk yield, fat yield and protein yield independent of stature.

To date, a total of 16 QTL affecting angularity have been identified [29] on 11 autosomes. A QTL for angularity in a Dutch Holstein population was detected on BTA11 at 9.70-10.66 Mb (19.4 cM) [40], but far away from our signal at 87.3-88.0 Mb.

Schulman et al. [41] reported a QTL affecting somatic cell score at between 28.8 and 30.1 Mb (35.1 cM) on BTA24 in a Finnish Ayrshire cattle population. Recently, another QTL for the same trait was detected between 30.1 and 43.0 Mb (35.5-48.8 cM) on the same chromosome in Danish Holstein cattle [42]. Our association peak at 31.1 Mb for somatic cell score was in the same region as these QTL and also contains a microRNA (*bta-mir-2380;* Additional file 5: Figure S4A), which is expressed upon viral infection [43].

Milking speed is a workability trait that is very important to dairy producers. Cows that milk out fast require less labour in the milking hall. However, fast-milking cows may be at increased risk for mastitis [44]. The significantly associated SNPs in the peak on BTA6 for milking speed found in this study overlapped with a previously identified QTL in three French dairy cattle breeds [45]. Interestingly, the association signal is located close to *IL8* (Additional file 5: Figure S4B), a known member of interleukin family. Further explorations of the relationship between the SNP polymorphism of *IL8*, milking speed and immune-response could potentially provide new insights to the biological mechanisms underlying this trait.

## Conclusions

Here we report the results from a GWAS analysis of nine production, fertility, conformation, udder health and workability traits using data from the international breeding evaluation program for Brown Swiss cattle. 74 genome-wide significant SNPs were found to be associated with one or multiple traits using an analysis based on a linear mixed model with genomic control. A strong, pleiotropic locus affecting stature, milk yield, fat yield, protein yield, lactating cow’s ability to recycle after calving and body depth was found on BTA25. Furthermore, particularly interesting signals were found on BTA11 for angularity, BTA 24 for somatic cell score and BTA6 for milking speed. Most of these signals overlapped with earlier reported QTL for related traits in dairy and beef cattle. Several known functional candidate genes could also be identified in these regions. Our study shows the usefulness of data from international breeding evaluation for identifying genetic variants associated with complex traits and that the overlap between association and QTL signals is apparently large in cattle. Further replication studies, as well as functional dissection of the molecular mechanisms underlying the reported signals, are needed to fully understand the complexity of trait regulation. But this study provides a first important step along this path.

## Methods

### Animal population

Genotypes and national estimated breeding values (EBVs) for a large number of Brown Swiss bulls are routinely delivered to Interbull Centre as part of the international breeding evaluation program for dairy cattle [46]. The national EBVs are then used to calculate an international EBV for each bull that could be used in selection of sires in Brown Swiss cattle breeding programs across the world. At present, the information of 7038 Brown Swiss bulls is available at Interbull Centre. We chose to only use the progeny tested proven bulls in our genome-wide association study, as national genetic evaluation for different traits have different starting points, and different traits were measured at different time in the process of progeny testing. The sample sizes were different for the traits (n = 2061-5043; Table 1).

### Adjustment of EBV

As a bull’s breeding value (here international EBVs) includes pedigree information, there is a risk that SNP could be significantly associated with the trait due to the pedigree information rather than phenotype. Thus, deregressed solutions removing the contribution of information from relatives were calculated for each bull. The equation for de-regression [47] is as follows:

$$DEBV=PA+\left(EBV-PA\right)/REL_{\mathrm{dau}}.$$

Where *DEBV* is de-regressed EBV, *PA* represents parent average, EBV is estimated breeding value, and *REL*_*dau*_ is reliability from daughters. This conversion analysis was done using the R package [48]. The bulls used in this study are elite bulls whose EBV has high reliability. The distribution of reliability values has a strong kurtosis and it was therefore deemed that the use of reliability values to weight the DEBV would not improve the results.

### Genotype quality control

Genotype information was initially available for 44,826 SNPs on the Illumina Bovine SNP50K Beadchip. We applied the following quality control of these SNPs before conducting statistical analysis: SNPs were discarded if i) its call rate was less than 90%, ii) if its minor allele frequency (MAF) was less than 2% or iii) it departed from Hardy-Weinberg equilibrium at a threshold of *p <* 0.01. Individuals were also excluded from the analysis if they had more than 10% missing genotypes. There were slight differences in the outcome of the quality control between the traits as the sample of individuals for the traits differed slightly for the reason described above.

### Principle component analysis

To examine whether population stratification was a problem in our data, principle component analysis (PCA) was carried out using the GCTA software [49], which implement the method proposed by Price et al. [28].

### Linear mixed model GWAS analyses accounting for confounding

To analyze this data, where familial confounding existed, a linear mixed model implemented in the program EMMAX [26] was used. The PLINK software [50] was used to edit files before the EMMAX analysis was run. The model implemented is

Y=Xβ+u+ε

Where **Y** denotes the vector of deregressed EBVs (DEBVs), *X* is the vector of genotypes at the locus being tested, *β* is the additive fixed effect attributed to the locus, and *ε* is the vector of residual error with *ε ~ N* (0, *Iσ*^*2*^_*e*_), and *u* is the vector of the background polygenic effects with *u ~ N* (0, *Gσ*^*2*^_*u*_).

The kinship matrix, *G,* describes the genome-wide relatedness between the individuals and is estimated once based on the identity-in-state (IBS) of the genotyped markers. The parameters of the model *σ*^*2*^_*u*_ and *σ*^*2*^_*e*_ are estimated using restricted maximum likelihood (REML) for each SNP. Generalized least squares (GLS) is employed to estimate the effect *β* and an F-test test to test the null hypothesis *H*_*0*_: *β =0*.

Significance testing was based on Bonferroni corrected significance thresholds correcting for the number of SNP loci tested. Genomic control [21] was also used in order to correct for the weak inflation that still existed.

### Candidate gene identification

Locations of SNPs and gene clusters were identified based on the Bovine genome NCBI build 6.1 and ENSEMBL [34,35].

## Abbrevations

GWAS: Genome-wide association study; PCA: Principle component analysis; LD: Linkage disequilibrium; SNP: Single nucleotide polymorphism; Bp: Base pairs; Kb: Kilo base pairs; Mb: Mega base pairs; MAF: Minor allele frequency; cM: CentiMorgan; QTL: Quantitative trait locus; BTAN: *Bos taurus* chromosome N; EBVs: Estimated breeding values; DEBVs: Deregressed estimated breeding values; MY: Milk yield; FY: Fat yield; PY: Protein yield; CRC: Lactating cow’s ability to recycle after calving; ANG: Angularity; BDE: Body depth; STA: Stature; SCS: Milk somatic cell; MSP: Milking speed.

## Competing interests

The authors declare no competing interests.

## Authors’ contributions

ÖC and HJ initiated and planned the study. JG implemented the data analysis. JG drafted the manuscript and all authors together wrote the final paper. All authors read and approved the final manuscript.

## Supplementary Material

Additional file 1 **Figure S1.** Principal component analysis of the total sample of 7038 bulls. Plot of the first two principal components (PC1 and PC2) of each individual based on SNP information to evaluate the extent of population structure. Click here for file

Additional file 2 **FigureS2.** Manhattan plots for eight traits based on a linear mixed model and genomic control. Manhattan plot showing the association of the traits to BTA1-29. The chromosomes are plotted separately by color. The *P*-values of the association were after genomic control transformed to –log10 (*P*-values). The horizontal dashed line indicates the genome-wide Bonferroni-corrected significance level. Included traits are MY (milk yield), FY (fat yield), PY (protein yield), CRC (lactating cow’s ability to recycle after calving), ANG (angularity), BDE (body depth), SCS (milk somatic cell count) and MSP (milking speed). Click here for file

Additional file 3 **FigureS3.** Quantile-quantile (Q-Q) plots of *P*-values of SNPs from EMMAX for the nine analyzed traits The blue dots represent the association *P*-values. The dark line denotes the expected pattern under the null hypothesis, whereas the red line is the observed pattern. Deviations between the two lines indicate how the test statistics of loci deviate from the null hypothesis. MY: milk yield, FY: fat yield, PY: protein yield, CRC: lactating cow’s ability to recycle after calving, ANG: angularity, BDE: body depth, STA: stature, SCS: milk somatic cell count, MSP: milking speed. Click here for file

Additional file 4 **Table S1.** Genome-wide significant SNP effects for each of the nine analyzed traits. Click here for file

Additional file 5 **Figure S4.** 1 Mb gene clusters surrounding the association signals for somatic cell score on BTA24 and milking speed on BTA6. Gene clusters in 1 Mb regions surrounding the main association signals for A) somatic cell score on BTA24 and B) milking speed on BTA6. Click here for file

Additional file 6 **Figure S5.** The association of milk yield to BTA25 with and without stature as a covariate. The *p*-values for the association of milk yield to BTA25 was obtained using a linear mixed model and genomic control and then transformed to –log10 (*P*-values). The horizontal dashed line indicates the genome-wide significance level (i.e. corrected for testing all markers on BTA1-29). Additional file 5: Figure S5A shows the result of the analysis without and 5B the analysis with the stature phenotype as a covariate in the linear model. Click here for file

Additional file 7 **Figure S6.** The association of fat yield to BTA25 with and without stature as a covariate. The *p*-values for the association of fat yield to BTA25 was obtained using a linear mixed model and genomic control and then transformed to –log10 (*P*-values). The horizontal dashed line indicates the genome-wide significance level (i.e. corrected for testing all markers on BTA1-29). Additional file 6: Figure S6A shows the result of the analysis without and 6B the analysis with the stature phenotype as a covariate in the linear model. Click here for file
