# Supplementary figures and images for: A genome-wide association study using international breeding-evaluation data identifies major loci affecting production traits and stature in the Brown Swiss cattle breed
Source: BMC Genet. 2012 Oct 2;13:82. doi: 10.1186/1471-2156-13-82 (PMC3548702; doi:10.1186/1471-2156-13-82)

## Slide 1
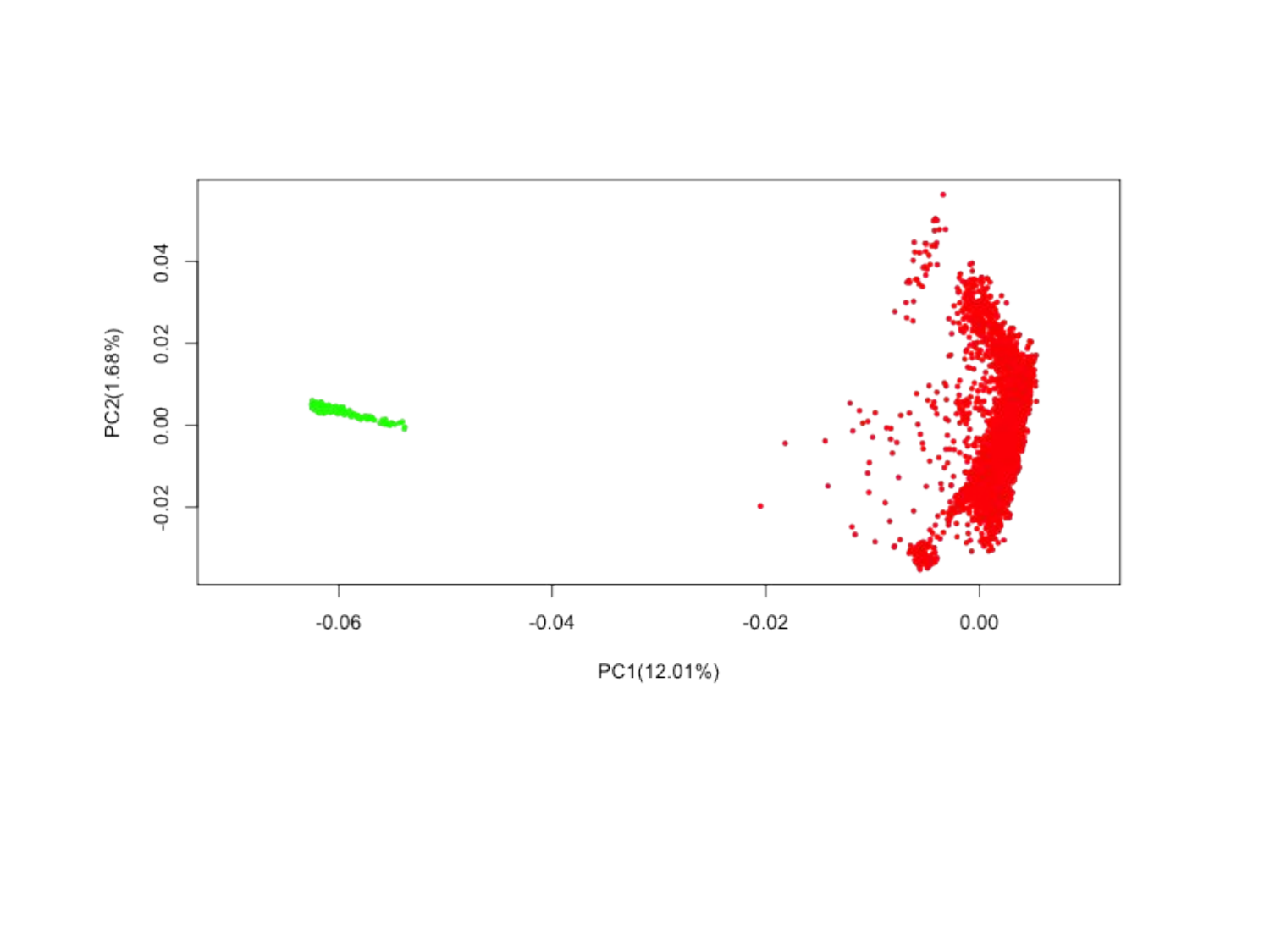

Supplement: Additional file 1 — Figure S1. Principal component analysis of the total sample of 7038 bulls. Plot of the first two principal components (PC1 and PC2) of each individual based on SNP information to evaluate the extent of population structure. [file 1471-2156-13-82-S1.pptx]

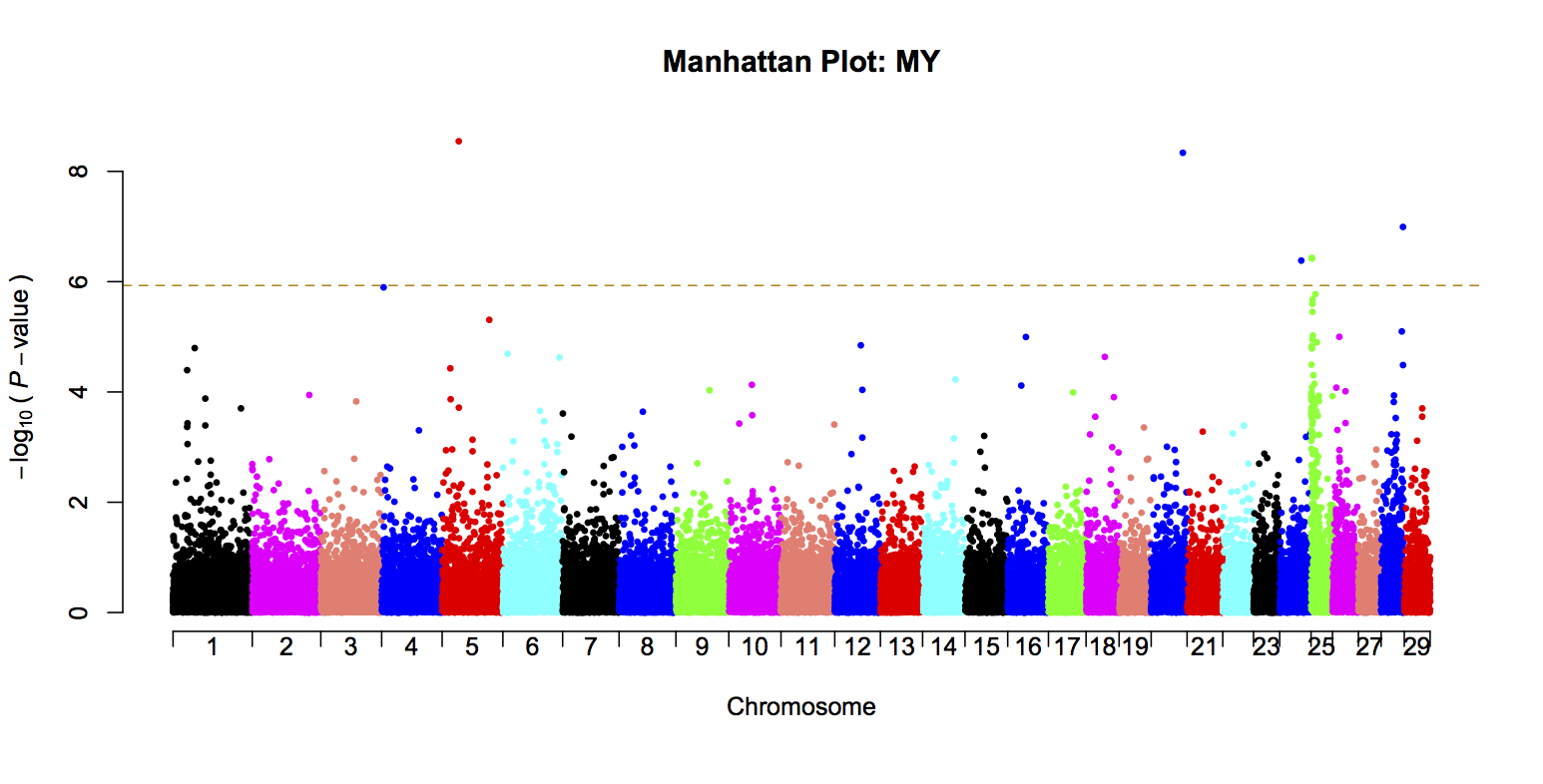


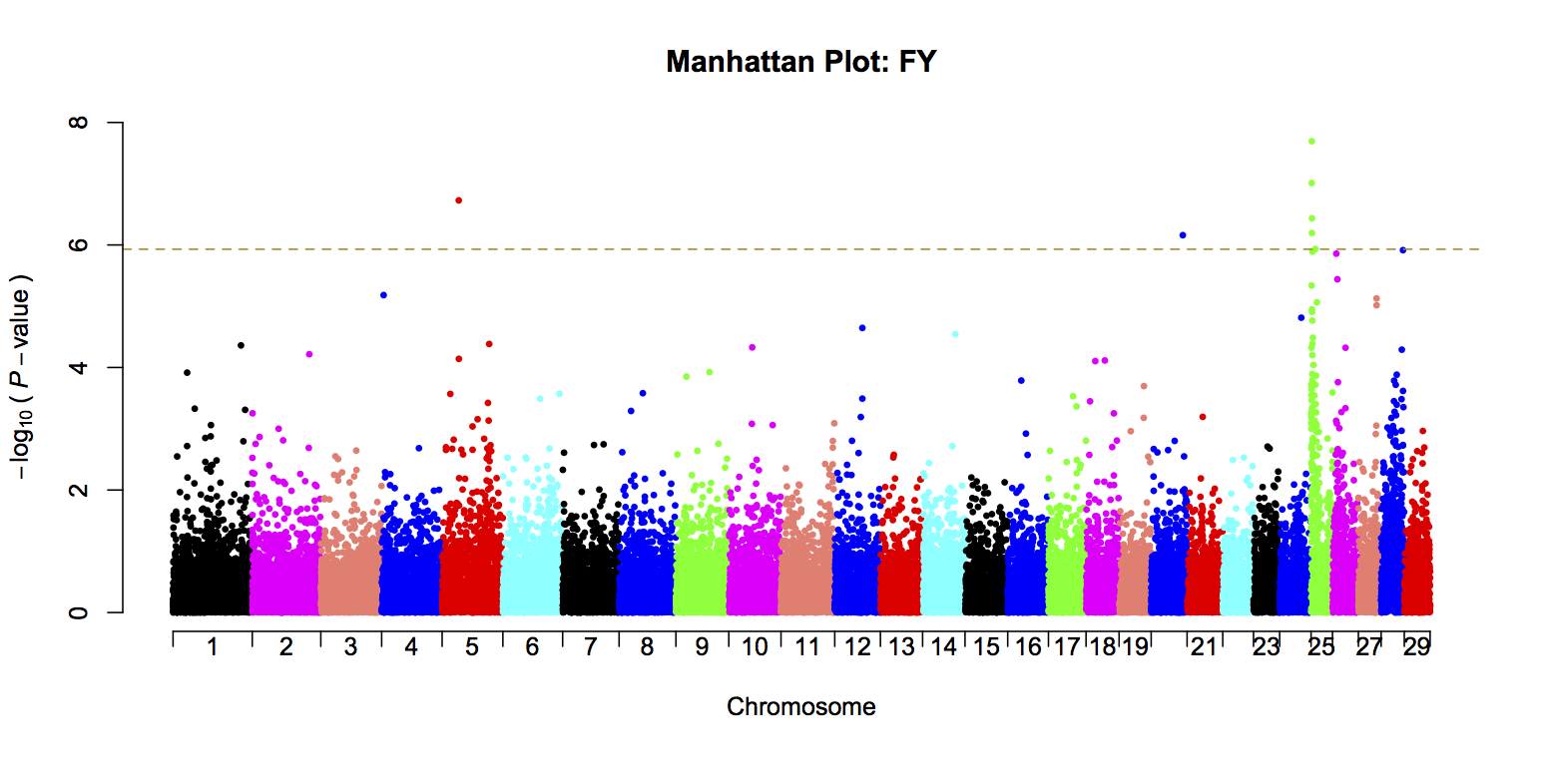


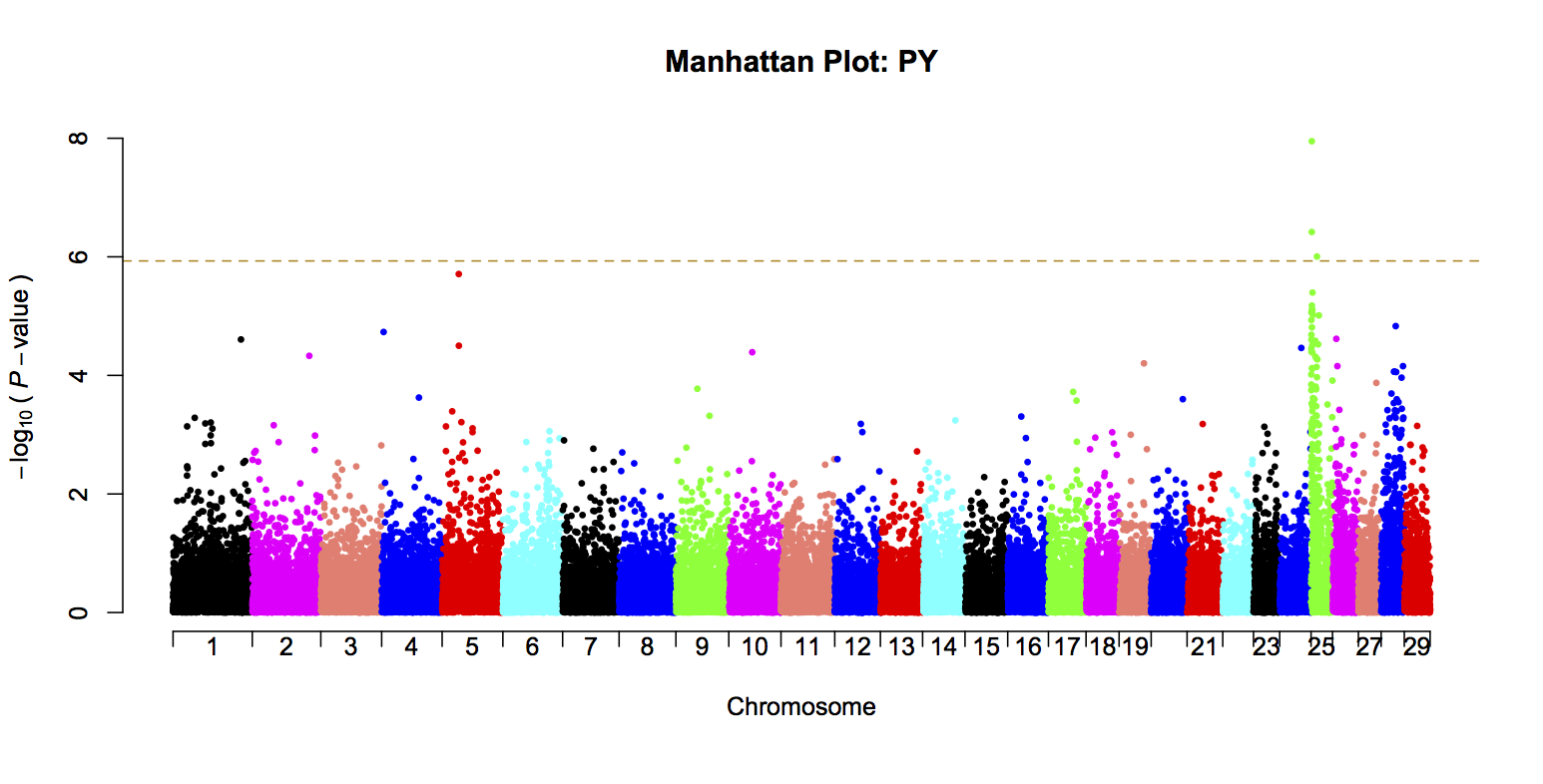


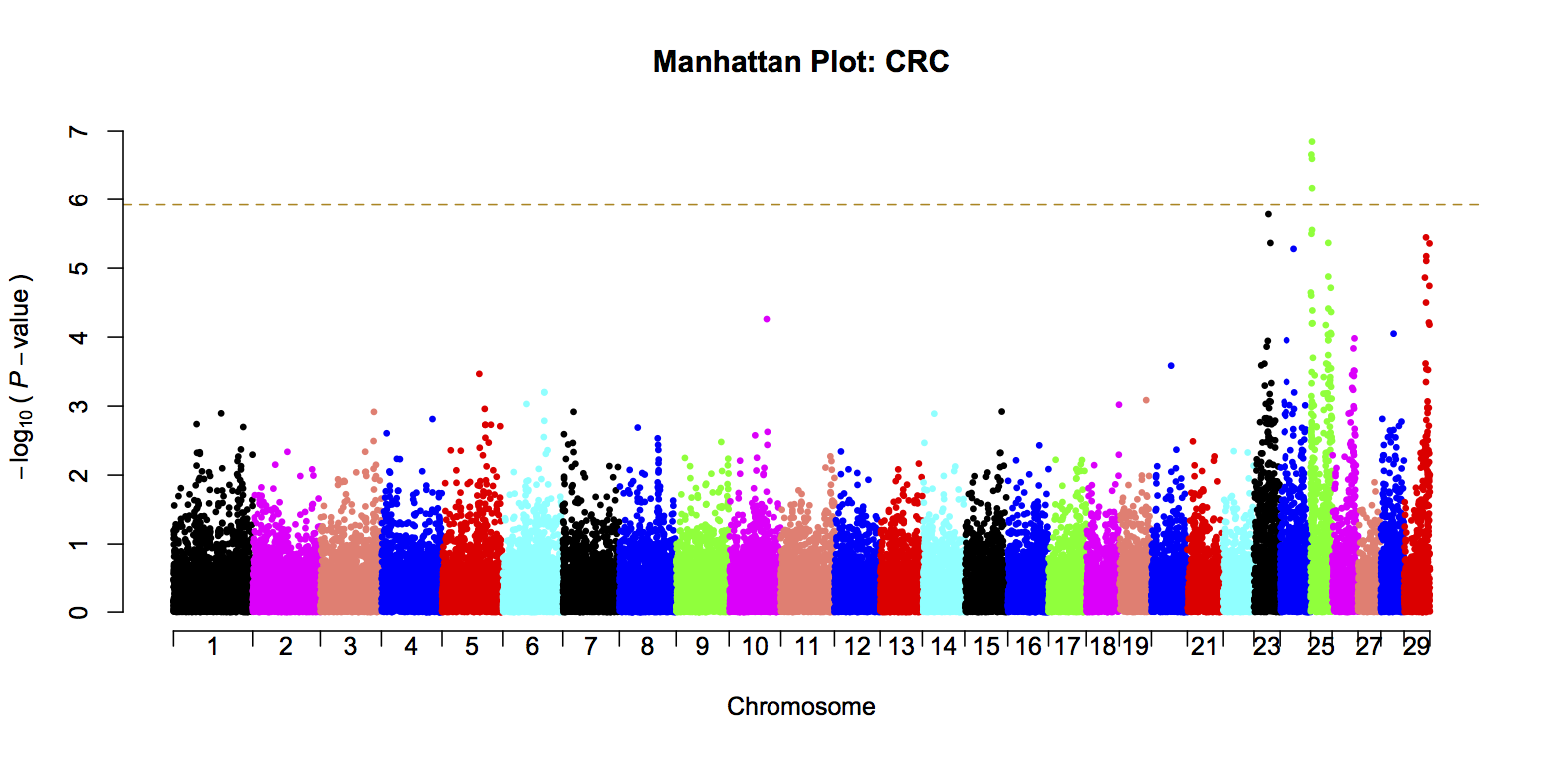


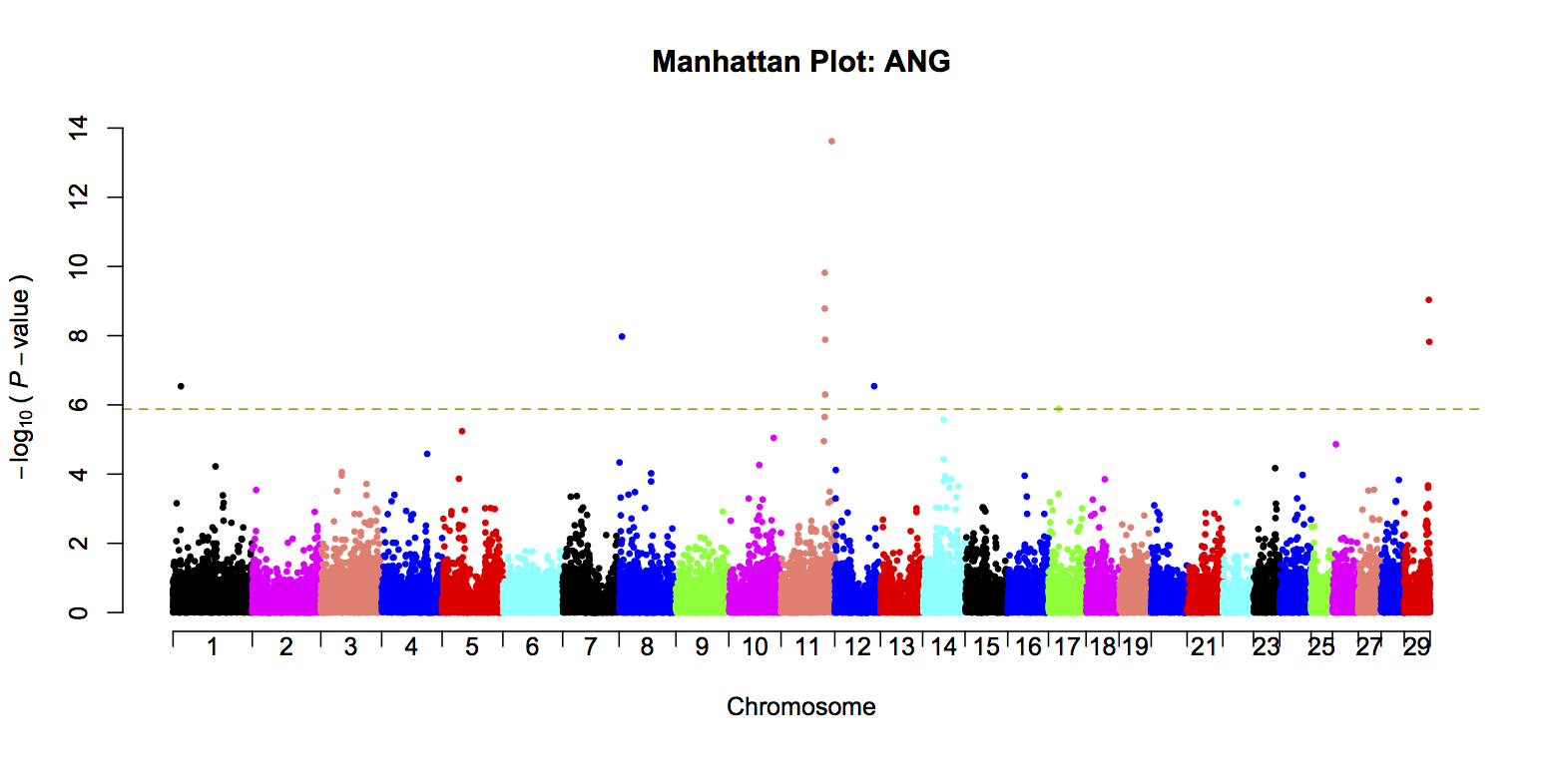


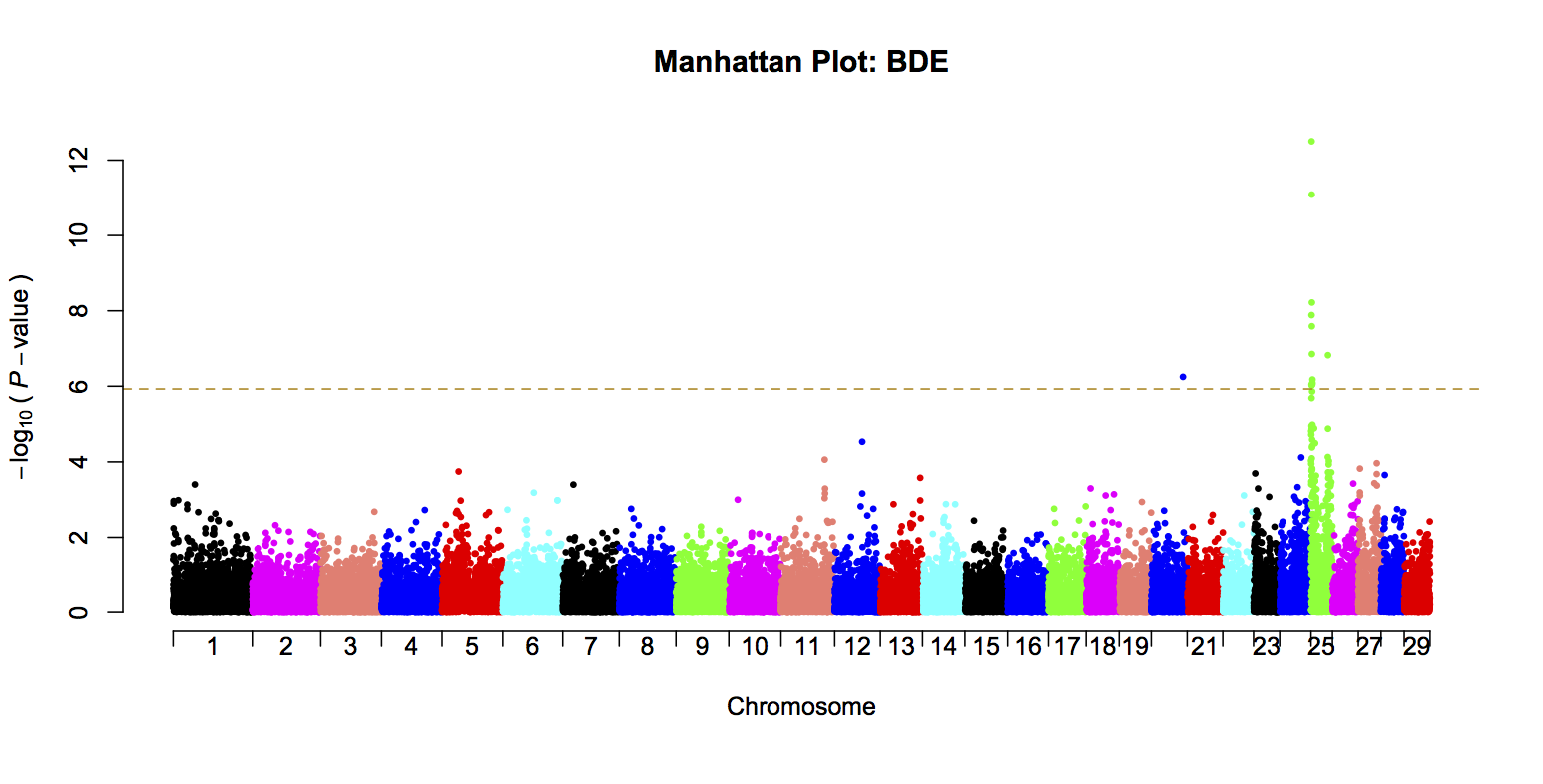


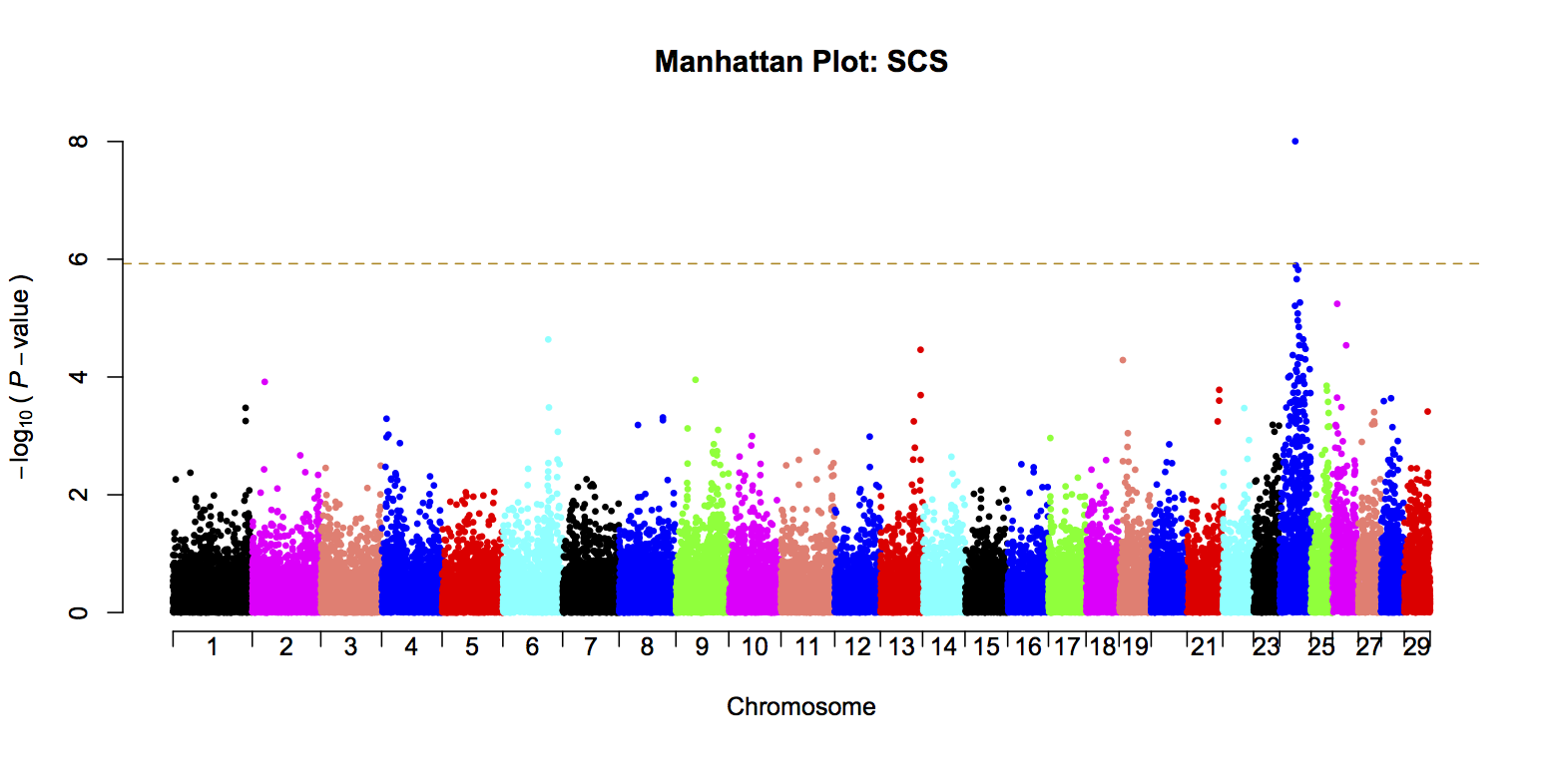


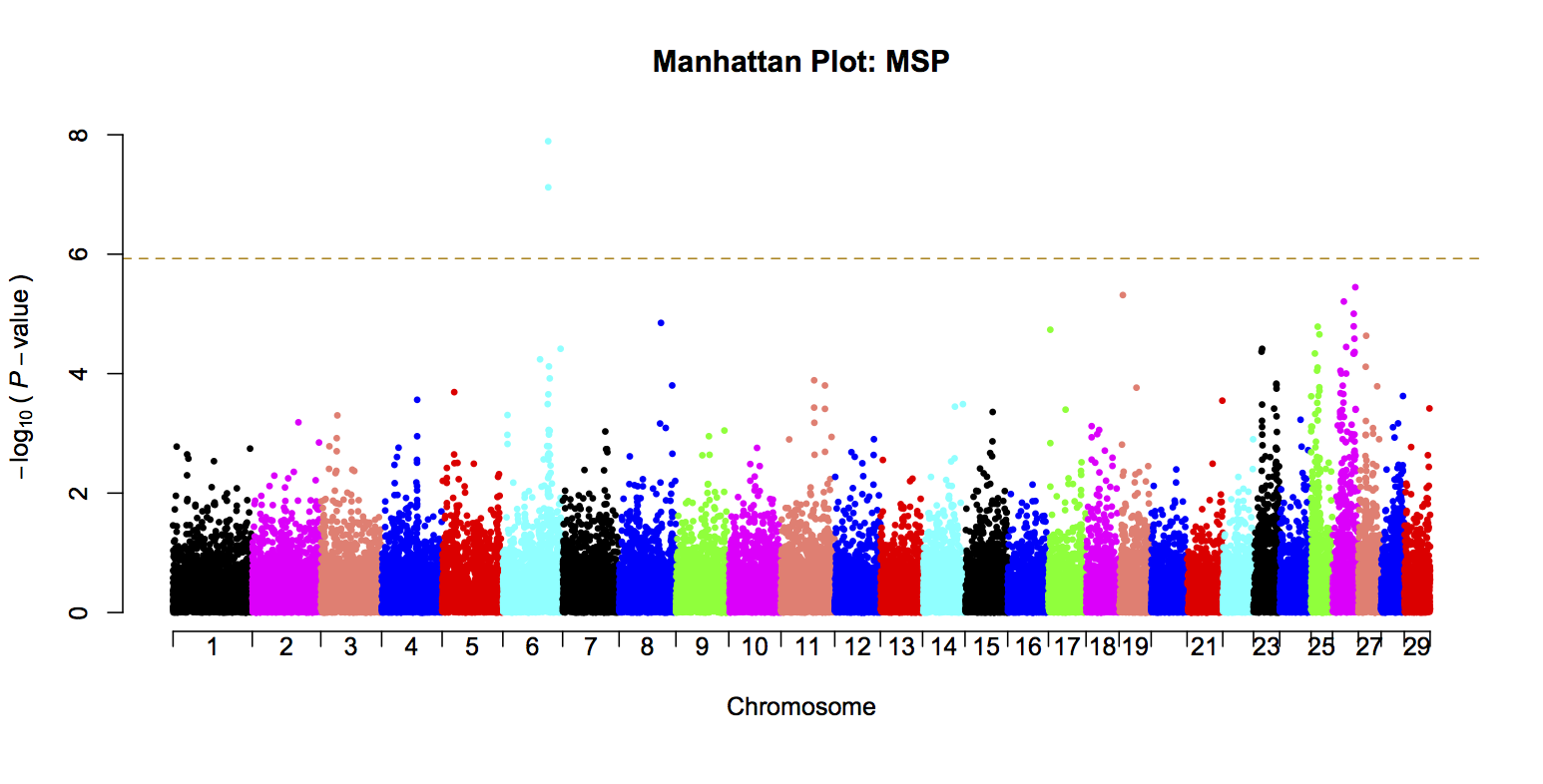

Supplement: Additional file 2 — FigureS2. Manhattan plots for eight traits based on a linear mixed model and genomic control. Manhattan plot showing the association of the traits to BTA1-29. The chromosomes are plotted separately by color. The P-values of the association were after genomic control transformed to –log10 (P-values). The horizontal dashed line indicates the genome-wide Bonferroni-corrected significance level. Included traits are MY (milk yield), FY (fat yield), PY (protein yield), CRC (lactating cow’s ability to recycle after calving), ANG (angularity), BDE (body depth), SCS (milk somatic cell count) and MSP (milking speed). [file 1471-2156-13-82-S2.docx]

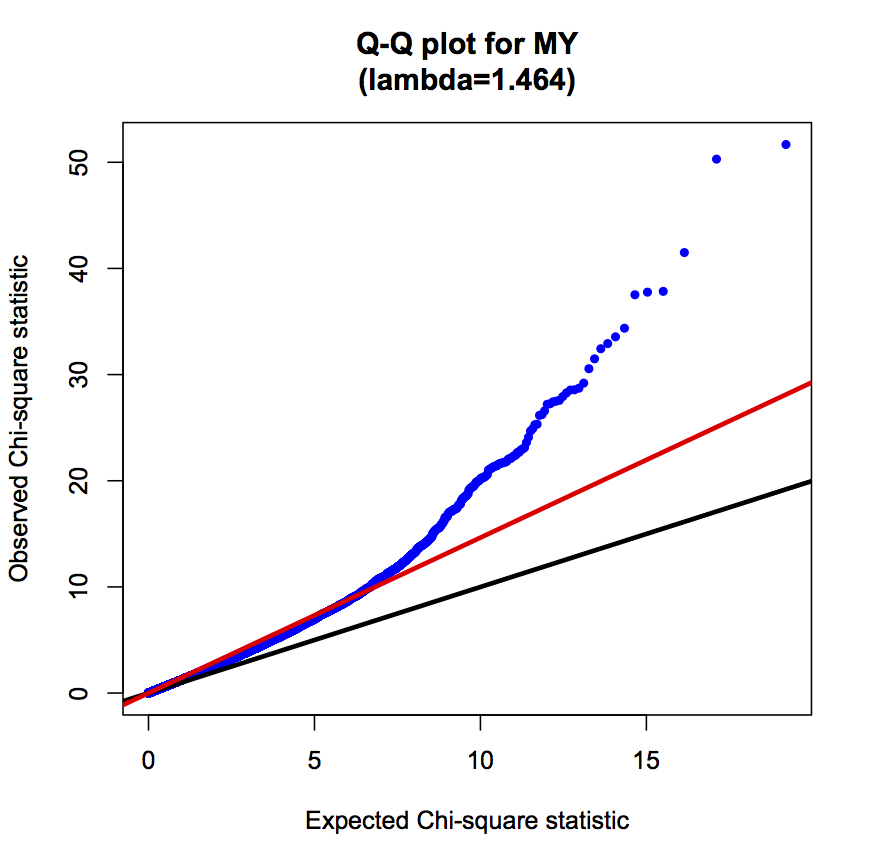


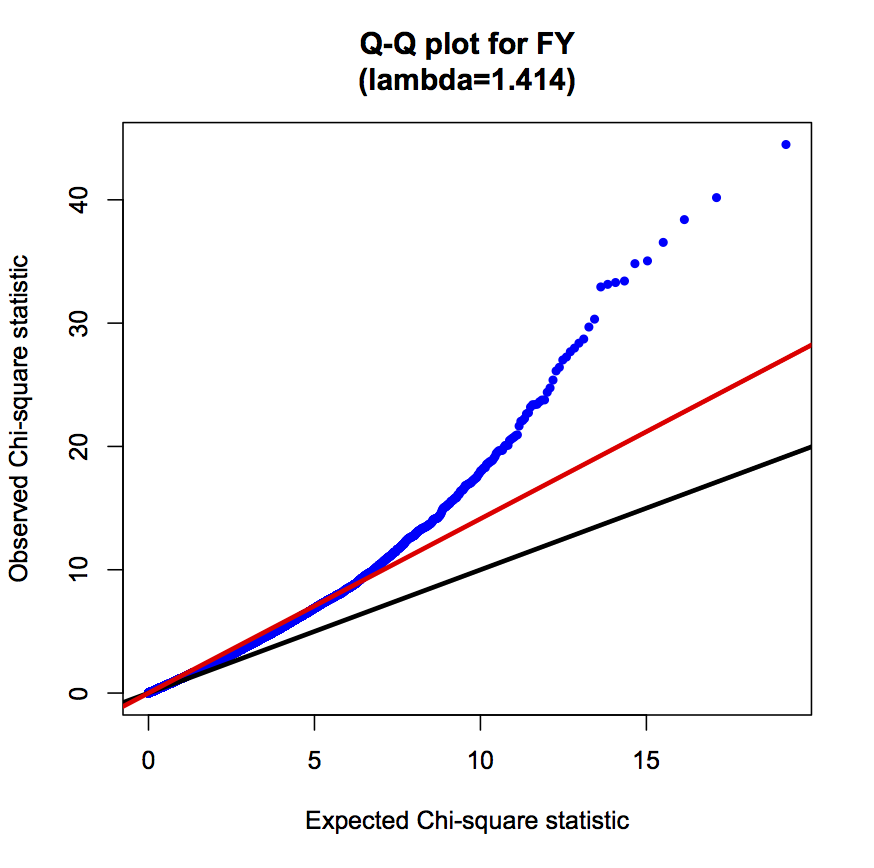


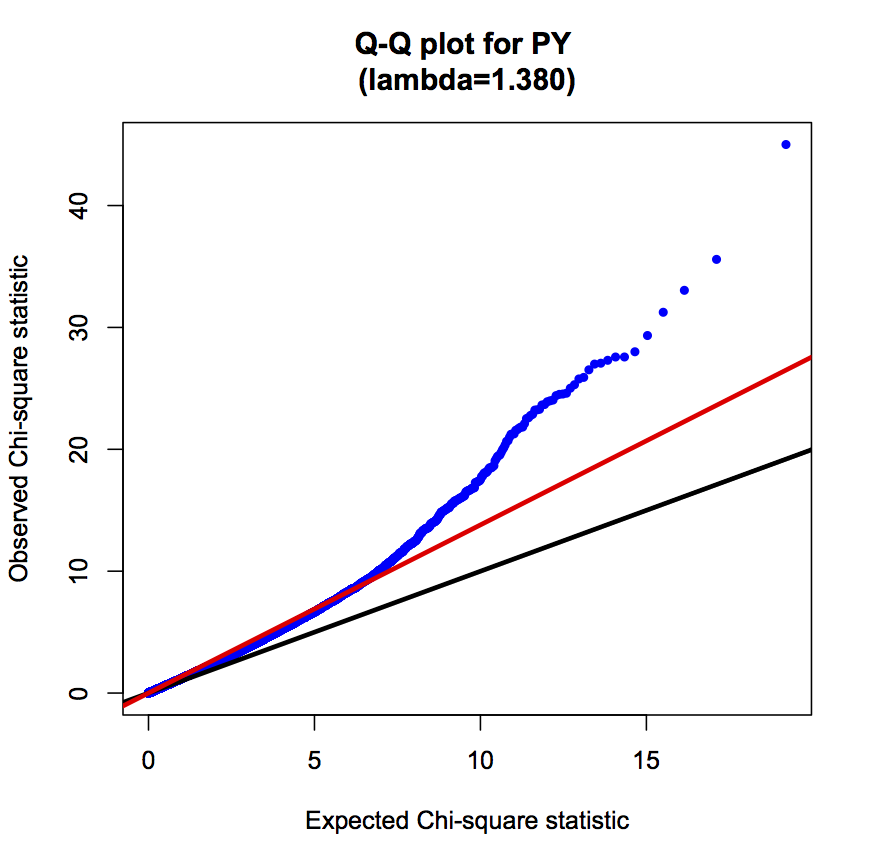


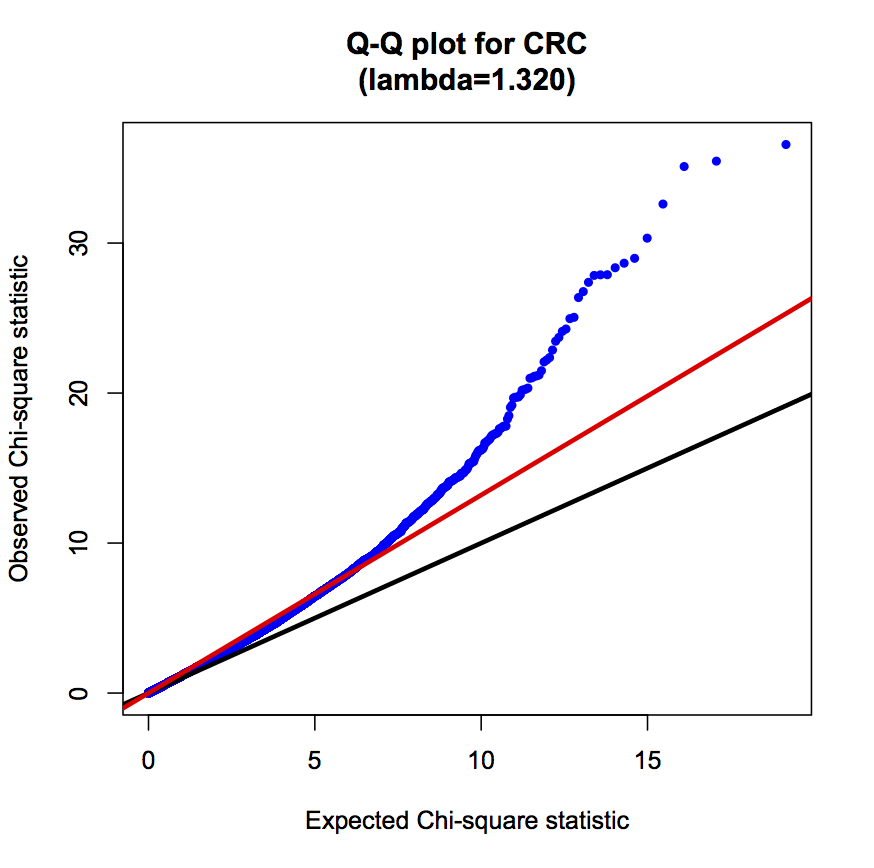


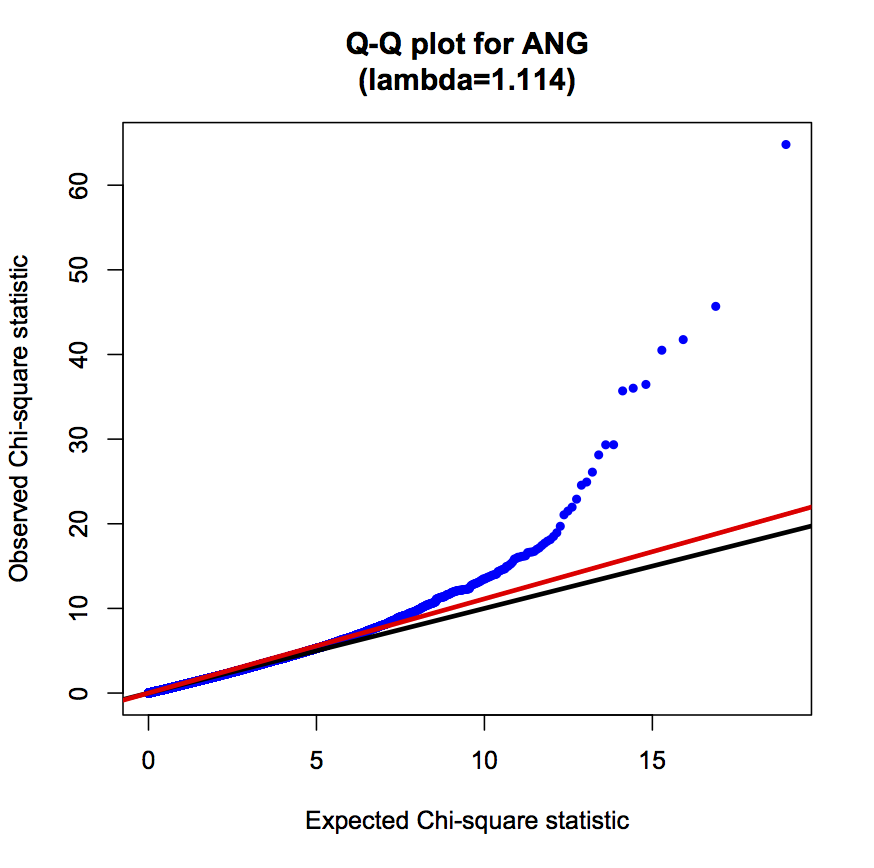


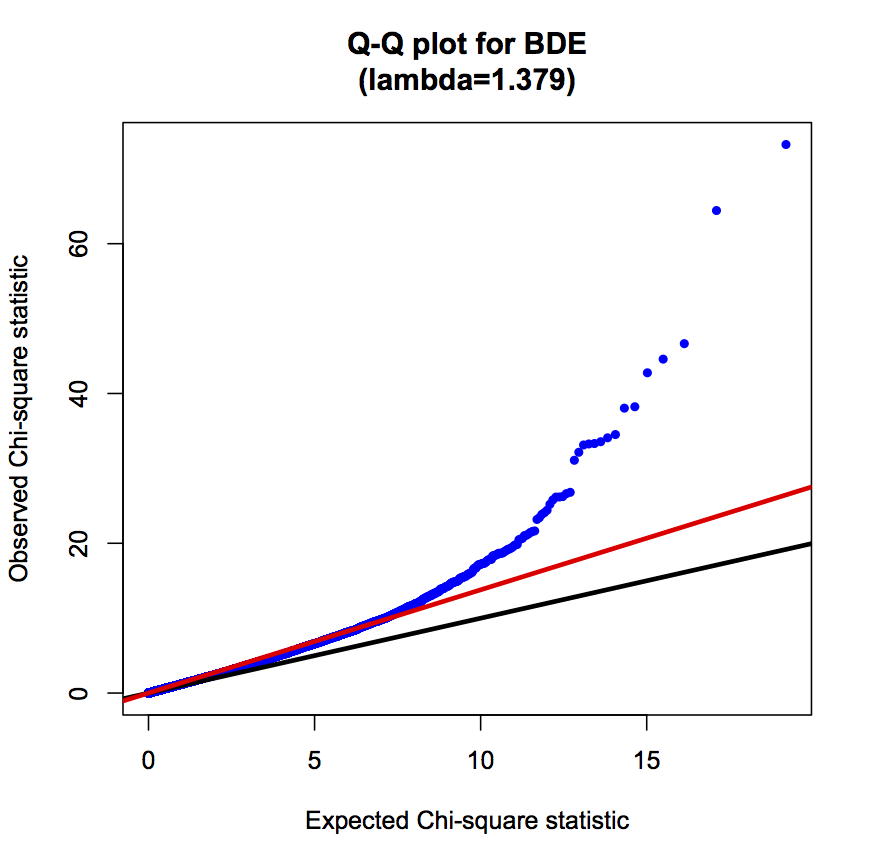


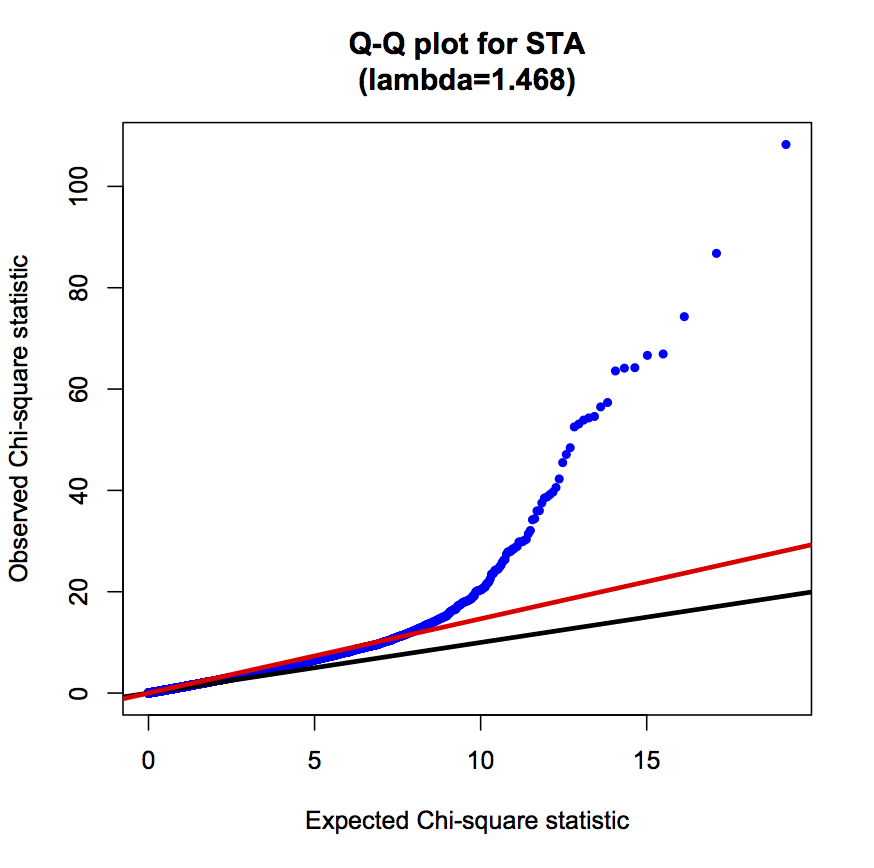


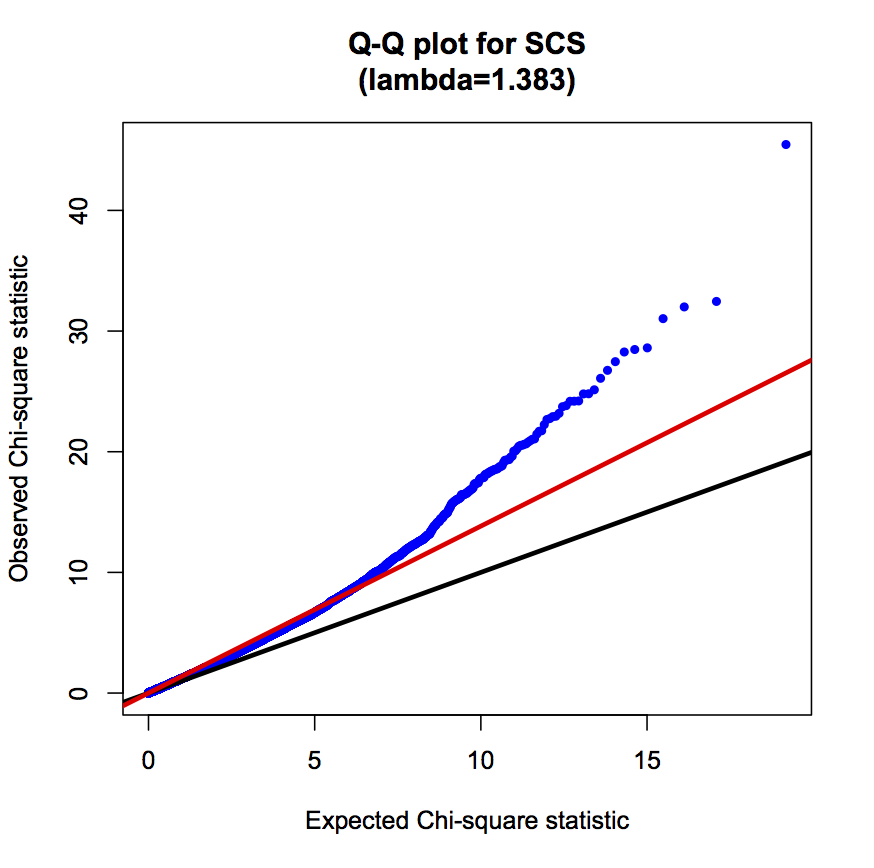


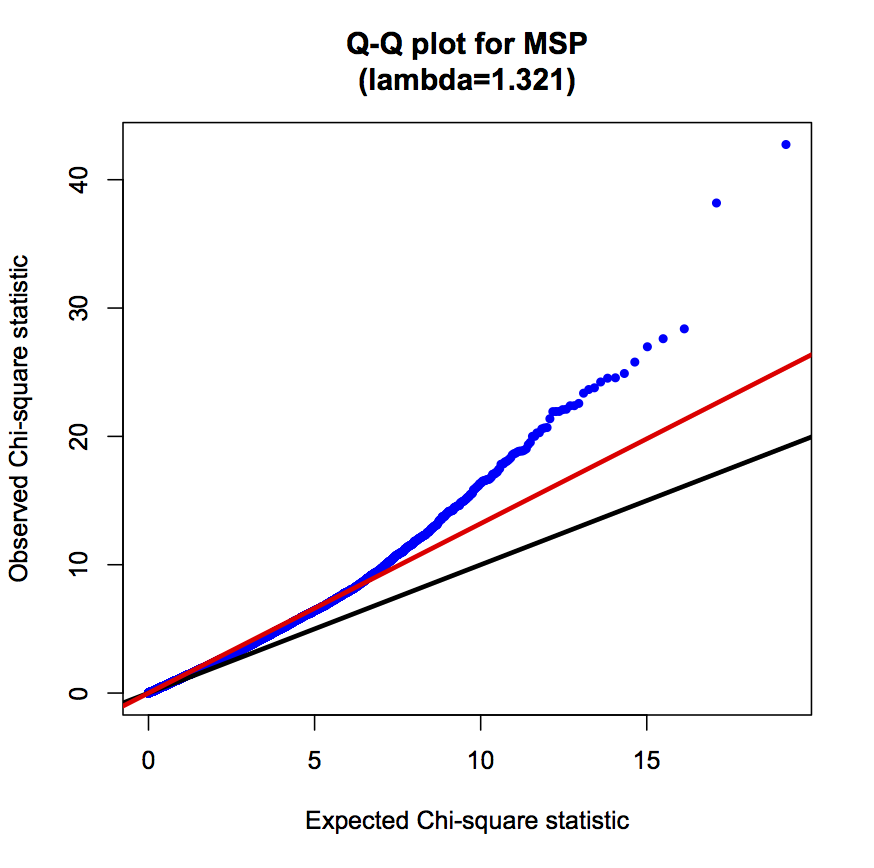

Supplement: Additional file 3 — FigureS3. Quantile-quantile (Q-Q) plots of P-values of SNPs from EMMAX for the nine analyzed traits The blue dots represent the association P-values. The dark line denotes the expected pattern under the null hypothesis, whereas the red line is the observed pattern. Deviations between the two lines indicate how the test statistics of loci deviate from the null hypothesis. MY: milk yield, FY: fat yield, PY: protein yield, CRC: lactating cow’s ability to recycle after calving, ANG: angularity, BDE: body depth, STA: stature, SCS: milk somatic cell count, MSP: milking speed. [file 1471-2156-13-82-S3.docx]

## Slide 1
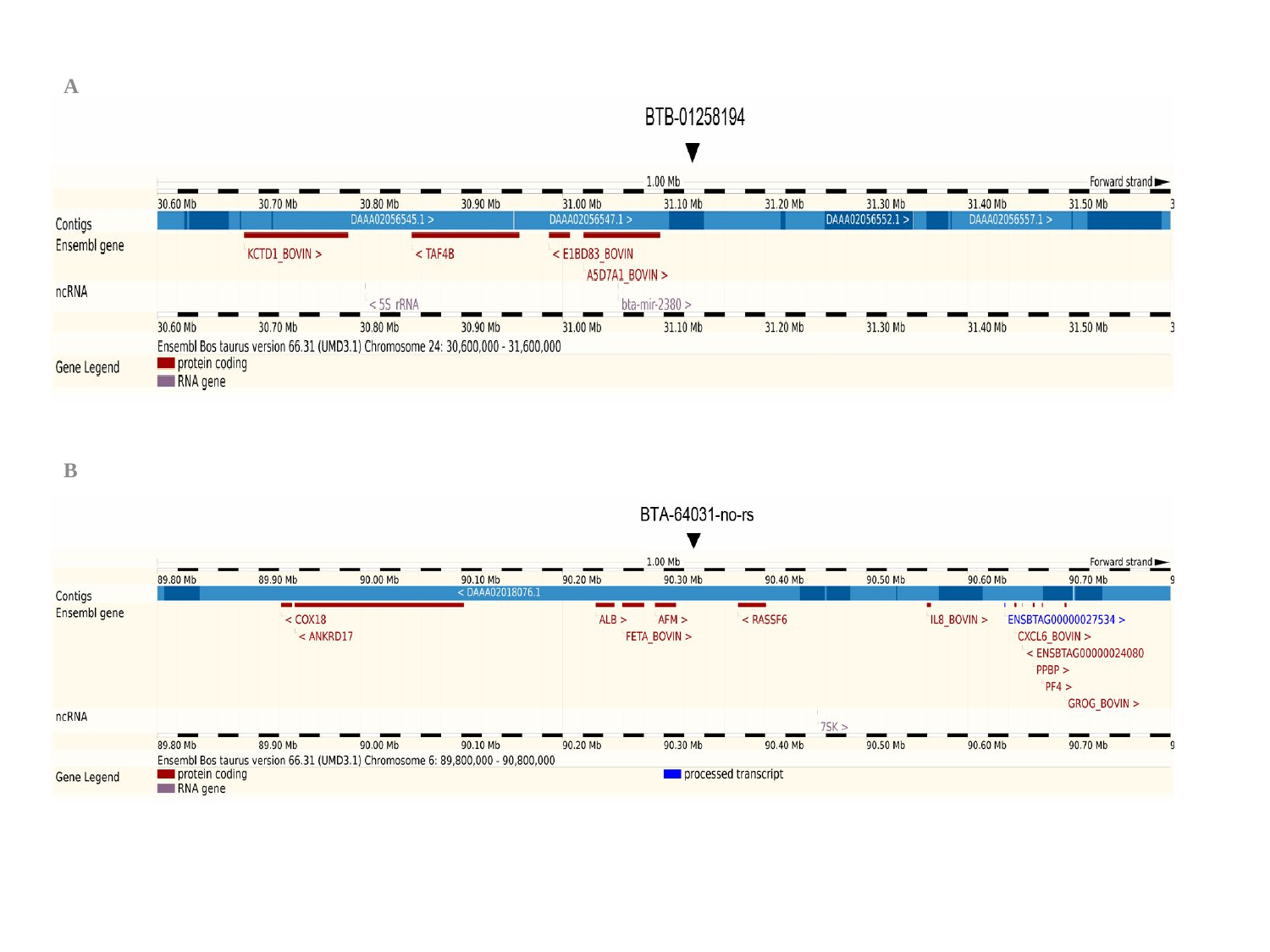

A
B

Supplement: Additional file 5 — Figure S4. 1 Mb gene clusters surrounding the association signals for somatic cell score on BTA24 and milking speed on BTA6. Gene clusters in 1 Mb regions surrounding the main association signals for A) somatic cell score on BTA24 and B) milking speed on BTA6. [file 1471-2156-13-82-S5.pptx]

## Slide 1
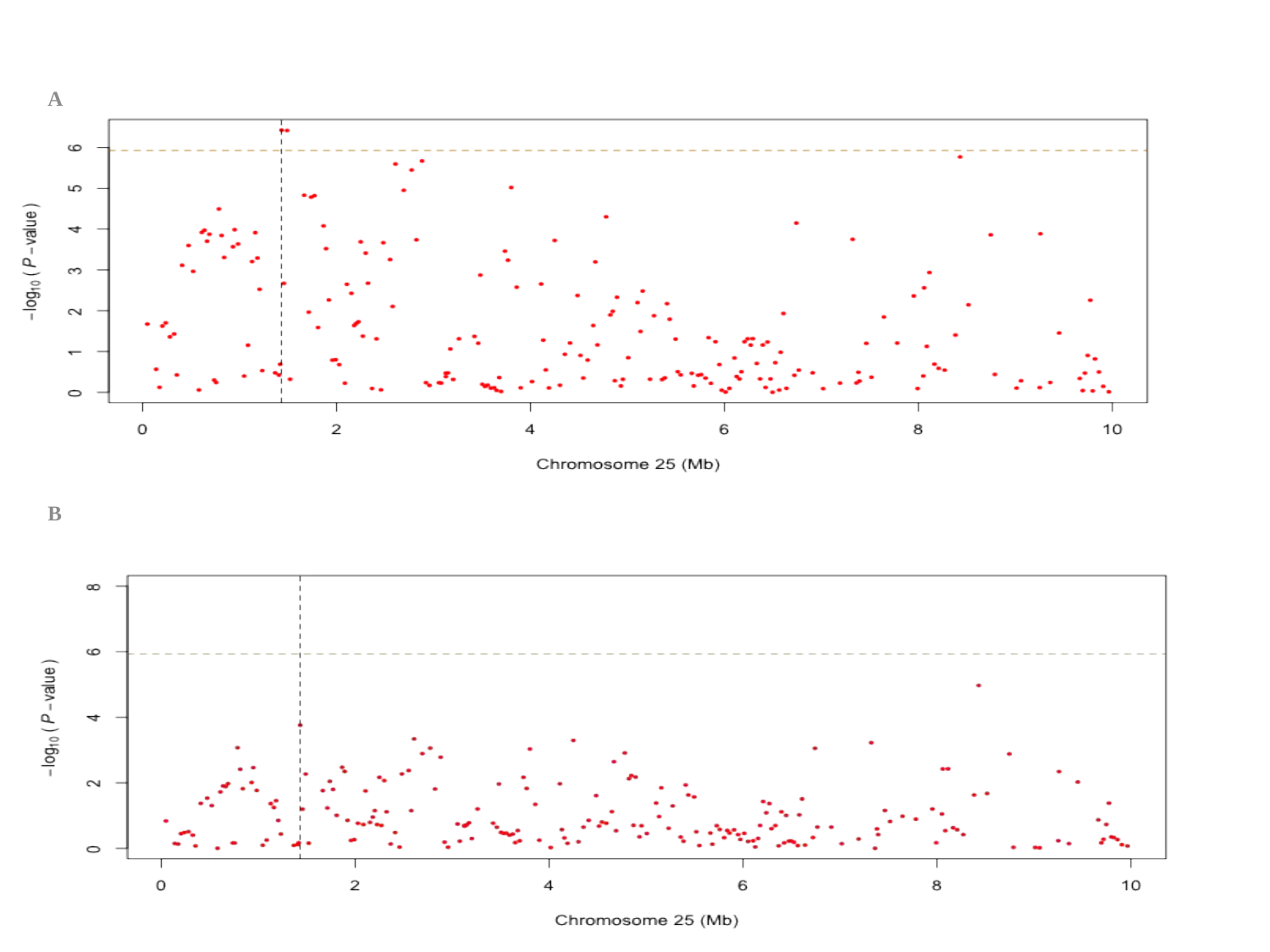

A
B

Supplement: Additional file 6 — Figure S5. The association of milk yield to BTA25 with and without stature as a covariate. The p-values for the association of milk yield to BTA25 was obtained using a linear mixed model and genomic control and then transformed to –log10 (P-values). The horizontal dashed line indicates the genome-wide significance level (i.e. corrected for testing all markers on BTA1-29). Additional file 5: Figure S5A shows the result of the analysis without and 5B the analysis with the stature phenotype as a covariate in the linear model. [file 1471-2156-13-82-S6.pptx]

## Slide 1
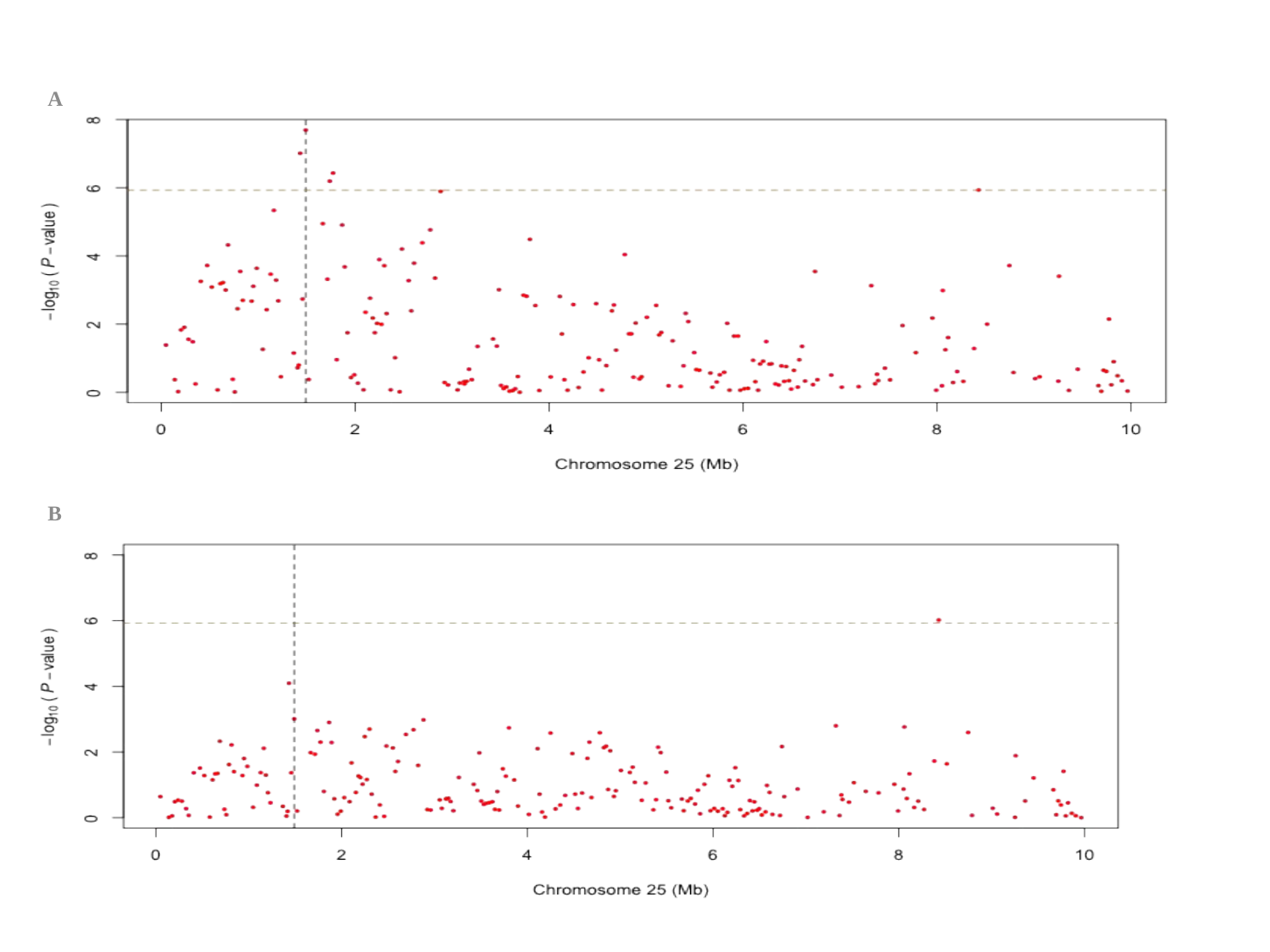

A
B

Supplement: Additional file 7 — Figure S6. The association of fat yield to BTA25 with and without stature as a covariate. The p-values for the association of fat yield to BTA25 was obtained using a linear mixed model and genomic control and then transformed to –log10 (P-values). The horizontal dashed line indicates the genome-wide significance level (i.e. corrected for testing all markers on BTA1-29). Additional file 6: Figure S6A shows the result of the analysis without and 6B the analysis with the stature phenotype as a covariate in the linear model. [file 1471-2156-13-82-S7.pptx]
