# Supplementary material for: A genome-wide association study using international breeding-evaluation data identifies major loci affecting production traits and stature in the Brown Swiss cattle breed
Source: BMC Genet. 2012 Oct 2;13:82. doi: 10.1186/1471-2156-13-82 (PMC3548702; doi:10.1186/1471-2156-13-82)
Supplement: Additional file 4 — Table S1. Genome-wide significant SNP effects for each of the nine analyzed traits. [file 1471-2156-13-82-S4.docx]

**Table S1: genome-wide significant SNP effects for each of 9 traits**

**Table S1.1: genome-wide significant SNP effects for milk yield**

| SNP | BTA | Position (bp) | Effect | MAF | *P*-value |
| --- | --- | --- | --- | --- | --- |
| Hapmap47184-BTA-114107 | 5 | 33173961 | 0.456 | 0.041 | 2.8E-09 |
| Hapmap48796-BTA-51083 | 20 | 63120443 | 0.513 | 0.033 | 4.6 E-09 |
| ARS-BFGL-NGS-56044 | 24 | 43170091 | 0.336 | 0.040 | 4.1 E-07 |
| Hapmap33541-BTC-016426 | 25 | 1431881 | 0.114 | 0.205 | 3.7 E-07 |
| ARS-BFGL-NGS-3562 | 25 | 1489008 | 0.098 | 0.186 | 3.8 E-07 |
| ARS-BFGL-NGS-102618 | 28 | 43609377 | 0.338 | 0.051 | 1.0 E-07 |

SNP, single nucleotide polymorphism; BTA, *Bos Taurus* Autosome; Effect, fixed effect of the SNP estimated from EMMAX in the standard deviation unit of milk yield; MAF, Minor Allele Frequency; *P*-value, *P*-value from EMMAX after Genomic control

**Table S1.2: genome-wide significant SNP effects for fat yield**

| SNP | BTA | Position (bp) | Effect | MAF | *P*-value |
| --- | --- | --- | --- | --- | --- |
| Hapmap47184-BTA-114107 | 5 | 33173961 | 0.388 | 0.041 | 1.9 E-07 |
| Hapmap48796-BTA-51083 | 20 | 63120443 | 0.421 | 0.033 | 6.9 E-07 |
| Hapmap33541-BTC-016426 | 25 | 1431881 | 0.118 | 0.269 | 9.7 E-08 |
| ARS-BFGL-NGS-3562 | 25 | 1489008 | 0.106 | 0.465 | 2.0 E-08 |
| ARS-BFGL-NGS-8982 | 25 | 1737669 | 0.102 | 0.335 | 6.4 E-07 |
| ARS-BFGL-NGS-79851 | 25 | 1771425 | 0.101 | 0.314 | 3.7 E-07 |
| BTA-60656-no-rs | 25 | 8430452 | 0.299 | 0.033 | 1.2 E-06 |

SNP, single nucleotide polymorphism; BTA, *Bos Taurus* Autosome; Effect, fixed effect of the SNP estimated from EMMAX in the standard deviation unit of fat yield; MAF, Minor Allele Frequency; *P*-value, *P*-value from EMMAX after Genomic control

**Table S1.3: genome-wide significant SNP effects for protein yield**

| SNP | BTA | Position (bp) | Effect | MAF | *P*-value |
| --- | --- | --- | --- | --- | --- |
| Hapmap33541-BTC-016426 | 25 | 1431881 | 0.119 | 0.269 | 1.1E-08 |
| ARS-BFGL-NGS-3562 | 25 | 1489008 | 0.091 | 0.465 | 3.8E-07 |
| ARS-BFGL-BAC-2924 | 25 | 11760835 | 0.092 | 0.359 | 9.9E-07 |

SNP, single nucleotide polymorphism; BTA, *Bos Taurus* Autosome; Effect, fixed effect of the SNP estimated from EMMAX in the standard deviation unit of protein yield; MAF, Minor Allele Frequency; *P*-value, *P*-value from EMMAX after Genomic control

**Table S1.4: genome-wide significant SNP effects for lactating cow’s ability to recycle after calving**

| SNP | BTA | Position (bp) | Effect | MAF | *P*-value |
| --- | --- | --- | --- | --- | --- |
| ARS-BFGL-NGS-3562 | 25 | 1489008 | -0.135 | 0.465 | 2.2E-07 |
| 000048794 | 25 | 2691937 | -0.132 | 0.485 | 2.5E-07 |
| 000048796 | 25 | 2773521 | -0.135 | 0.497 | 1.4E-07 |
| 000048799 | 25 | 2880252 | -0.129 | 0.467 | 6.7E-07 |

SNP, single nucleotide polymorphism; BTA, *Bos Taurus* Autosome; Effect, fixed effect of the SNP estimated from EMMAX in the standard deviation unit of lactating cow’s ability to recycle after calving; MAF, Minor Allele Frequency; *P*-value, *P*-value from EMMAX after Genomic control

**Table S1.5: genome-wide significant SNP effects for angularity**

| SNP | BTA | Position (bp) | Effect | MAF | *P*-value |
| --- | --- | --- | --- | --- | --- |
| ARS-BFGL-NGS-109692 | 1 | 15796320 | 0.751 | 0.039 | 2. E-10 |
| BTB-00334209 | 8 | 5753035 | 0.460 | 0.104 | 1.1E-08 |
| BTA-110370-no-rs | 11 | 87257595 | 0.607 | 0.073 | 1.5E-10 |
| ARS-BFGL-NGS-114578 | 11 | 87279008 | 0.570 | 0.050 | 1.6E-09 |
| ARS-BFGL-NGS-41670 | 11 | 87972079 | 0.441 | 0.069 | 5.0E-07 |
| ARS-BFGL-BAC-16207 | 11 | 87999946 | 0.544 | 0.062 | 1.3E-08 |
| ARS-BFGL-NGS-12589 | 11 | 101136744 | 0.791 | 0.062 | 2.4E-14 |
| ARS-BFGL-NGS-32303 | 12 | 78540440 | 0.596 | 0.048 | 2.9E-07 |
| ARS-BFGL-BAC-35952 | 17 | 20430382 | 0.390 | 0.101 | 1.3E-06 |
| ARS-BFGL-NGS-10561 | 29 | 48915813 | 0.455 | 0.054 | 9.2E-10 |
| ARS-BFGL-NGS-102385 | 29 | 49779067 | -0.471 | 0.069 | 1.5E-08 |

SNP, single nucleotide polymorphism; BTA, *Bos Taurus* Autosome; Effect, fixed effect of the SNP estimated from EMMAX in the standard deviation unit of angularity; MAF, Minor Allele Frequency; *P*-value, *P*-value from EMMAX after Genomic control

**Table S1.6: genome-wide significant SNP effects for body depth**

| SNP | BTA | Position (bp) | Effect | MAF | *P*-value |
| --- | --- | --- | --- | --- | --- |
| Hapmap48796-BTA-51083 | 20 | 63120443 | -0.544 | 0.033 | 5.7E-07 |
| ARS-BFGL-NGS-61709 | 25 | 1086505 | -0.186 | 0.202 | 1.3E-08 |
| ARS-BFGL-NGS-111712 | 25 | 1160378 | -0.179 | 0.425 | 3.2E-13 |
| ARS-BFGL-BAC-43143 | 25 | 1184038 | -0.134 | 0.297 | 9.1E-07 |
| ARS-BFGL-BAC-44214 | 25 | 1404930 | -0.136 | 0.232 | 9.5E-07 |
| ARS-BFGL-NGS-3562 | 25 | 1489008 | 0.169 | 0.465 | 8.2E-12 |
| ARS-BFGL-NGS-15055 | 25 | 1665327 | -0.138 | 0.440 | 2.6E-08 |
| ARS-BFGL-NGS-8982 | 25 | 1737669 | 0.157 | 0.335 | 6.0E-09 |
| ARS-BFGL-NGS-79851 | 25 | 1771425 | -0.138 | 0.314 | 1.4E-07 |
| ARS-BFGL-NGS-12473 | 25 | 2691937 | 0.121 | 0.485 | 8.9E-07 |
| Hapmap26816-BTC-015808 | 25 | 2773521 | 0.121 | 0.497 | 8.1E-07 |
| ARS-BFGL-NGS-10250 | 25 | 2880252 | 0.124 | 0.467 | 6.7E-07 |
| ARS-BFGL-NGS-44485 | 25 | 33852697 | -0.126 | 0.439 | 1.5E-07 |

SNP, single nucleotide polymorphism; BTA, *Bos Taurus* Autosome; Effect, fixed effect of the SNP estimated from EMMAX in the standard deviation unit of body depth; MAF, Minor Allele Frequency; *P*-value, *P*-value from EMMAX after Genomic control

**Table S1.7: genome-wide significant SNP effects for stature**

| SNP | BTA | Position (bp) | Effect | MAF | *P*-value |
| --- | --- | --- | --- | --- | --- |
| BTB-00492401 | 12 | 47444560 | 0.805 | 0.026 | 1.5E-07 |
| ARS-BFGL-NGS-119643 | 25 | 324116 | -0.125 | 0.377 | 2.0E-07 |
| ARS-BFGL-BAC-46926 | 25 | 406196 | 0.144 | 0.444 | 1.4E-09 |
| ARS-BFGL-NGS-12001 | 25 | 472458 | 0.158 | 0.355 | 4.1E-10 |
| ARS-BFGL-NGS-20408 | 25 | 520071 | 0.142 | 0.463 | 1.8E-09 |
| ARS-BFGL-NGS-7427 | 25 | 609241 | -0.148 | 0.394 | 5.5E-10 |
| ARS-BFGL-NGS-14220 | 25 | 636274 | -0.146 | 0.371 | 1.1E-09 |
| ARS-BFGL-NGS-40627 | 25 | 664032 | -0.138 | 0.368 | 9.2E-09 |
| ARS-BFGL-NGS-108460 | 25 | 687508 | 0.149 | 0.794 | 8.0E-08 |
| ARS-BFGL-NGS-103099 | 25 | 1127441 | -0.146 | 0.286 | 1.5E-08 |
| ARS-BFGL-NGS-111712 | 25 | 1160378 | -0.166 | 0.425 | 1.1E-12 |
| ARS-BFGL-BAC-43143 | 25 | 1184038 | -0.144 | 0.297 | 2.6E-08 |
| Hapmap29768-BTC-016149 | 25 | 1205232 | -0.155 | 0.155 | 7.3E-07 |
| Hapmap33541-BTC-016426 | 25 | 1431881 | -0.141 | 0.269 | 2.4E-07 |
| Hapmap31901-BTC-016378 | 25 | 1456346 | -0.138 | 0.276 | 4.3E-07 |
| ARS-BFGL-NGS-3562 | 25 | 1489008 | 0.200 | 0.465 | 8.8E-18 |
| ARS-BFGL-NGS-15055 | 25 | 1665327 | -0.179 | 0.440 | 1.5E-14 |
| ARS-BFGL-NGS-8982 | 25 | 1737669 | 0.168 | 0.335 | 4.6E-11 |
| ARS-BFGL-NGS-79851 | 25 | 1771425 | -0.150 | 0.314 | 1.2E-09 |
| Hapmap22786-BTC-071372 | 25 | 1919606 | -0.136 | 0.375 | 2.2E-09 |
| ARS-BFGL-BAC-28144 | 25 | 2606575 | 0.185 | 0.135 | 3.0E-07 |
| ARS-BFGL-NGS-12473 | 25 | 2691937 | 0.153 | 0.485 | 3.8E-11 |
| Hapmap26816-BTC-015808 | 25 | 2773521 | 0.156 | 0.497 | 1.4E-11 |
| ARS-BFGL-NGS-8420 | 25 | 2823458 | 0.155 | 0.493 | 1.6E-11 |
| ARS-BFGL-NGS-10250 | 25 | 2880252 | 0.206 | 0.467 | 3.7E-11 |
| Hapmap26526-BTC-001269 | 25 | 5104091 | -0.120 | 0.324 | 7.4E-07 |
| ARS-BFGL-NGS-114786 | 25 | 7952738 | -0.126 | 0.305 | 2.8E-07 |

SNP, single nucleotide polymorphism; BTA, *Bos Taurus* Autosome; Effect, fixed effect of the SNP estimated from EMMAX in the standard deviation unit of stature; MAF, Minor Allele Frequency; *P*-value, *P*-value from EMMAX after Genomic control

**Table S1.8: genome-wide significant SNP effects for somatic cell score**

| SNP | BTA | Position (bp) | Effect | MAF | *P*-value |
| --- | --- | --- | --- | --- | --- |
| BTB-01258194 | 24 | 31133584 | -0.136 | 0.267 | 9.9E-07 |

SNP, single nucleotide polymorphism; BTA, *Bos Taurus* Autosome; Effect, fixed effect of the SNP estimated from EMMAX in the standard deviation unit of somatic cell score; MAF, Minor Allele Frequency; *P*-value, *P*-value from EMMAX after Genomic control

**Table S1.9: genome-wide significant SNP effects for milking speed**

| SNP | BTA | Position (bp) | Effect | MAF | *P*-value |
| --- | --- | --- | --- | --- | --- |
| BTA-64031-no-rs | 6 | 90325414 | -0.215 | 0.315 | 1.3E-08 |
| BTA-77009-no-rs | 6 | 90486780 | 0.202 | 0.184 | 7.6E-08 |

SNP, single nucleotide polymorphism; BTA, *Bos Taurus* Autosome; Effect, fixed effect of the SNP estimated from EMMAX in the standard deviation unit of milking speed; MAF, Minor Allele Frequency; *P*-value, *P*-value from EMMAX after Genomic control
